# Zinc prevents vaginal candidiasis by inhibiting expression of an inflammatory fungal protein

**DOI:** 10.1126/scitranslmed.adi3363

**Published:** 2023-12-06

**Authors:** Elena Roselletti, Eva Pericolini, Alexandre Nore, Peter Takacs, Bence Kozma, Arianna Sala, Francesco De Seta, Manola Comar, Jane Usher, Gordon D Brown, Duncan Wilson

**Affiliations:** 1 Medical Research Council Centre for Medical Mycology at The University of Exeter, University of Exeter, Geoffrey Pope Building Stocker Road, Exeter, UK, EX4 4QD; 2 Department of Surgical, Medical, Dental and Morphological Sciences with Interest in Transplant, Oncological and Regenerative Medicine, University of Modena and Reggio Emilia, Modena, Italy, 41125; 3 Department of Obstetrics and Gynecology, Faculty of Medicine, University of Debrecen, Debrecen, Hungary, 4032; 4 Division of Female Pelvic Medicine and Reconstructive Surgery, Department of Obstetrics and Gynecology, Eastern Virginia Medical School, Norfolk, VA, USA, 23507; 5 Department of Medical Sciences, University of Trieste, Institute for Maternal and Child Health-IRCCS, Burlo Garofolo, Trieste, Italy, 34137; 6 Unit of Advanced Microbiology Diagnosis and Translational Research, Institute for Maternal and Child Health—IRCCS “Burlo Garofolo”, University of Trieste, Trieste, Italy, 34137

## Abstract

*Candida* causes an estimated half billion cases of vulvovaginal candidiasis (VVC) every year. VVC is most commonly caused by *Candida albicans*, which in this setting triggers non-protective neutrophil infiltration, aggressive local inflammation, and symptomatic disease. Despite its prevalence, little is known about the molecular mechanisms underpinning the immunopathology of this fungal infection. In this study we describe the molecular determinant of VVC immunopathology and a potentially straightforward way to prevent disease. In response to zinc limitation, *C. albicans* releases a trace mineral binding molecule called Pra1 (pH-regulated antigen). Here we show that the *PRA1* gene is strongly upregulated during vaginal infections and that its expression positively correlated with levels of proinflammatory cytokines in women. Genetic deletion of *PRA1* prevented vaginal inflammation in mice and application of a zinc solution down-regulated expression of the gene and also blocked immunopathology. We also show that treatment of women suffering from recurrent vulvovaginal candidiasis with a zinc gel prevented reinfections. We have therefore identified a key mediator of symptomatic VVC, giving us an unparalleled opportunity to develop a new range of preventative measures for combatting this disease.

## Introduction

*Candida albicans* is a commensal member of the human oral, gastrointestinal, and urogenital microbiota. However, this yeast can also cause infections in humans. The most common type of *Candida* infection is vulvovaginal candidiasis (VVC, also known as thrush). VVC affects three quarters of women of childbearing age and ~7% of those affected suffer from recurrent infections (four or more vaginal infections per year). Together, this accounts for an estimated half billion infections every year, which can have an enormous impact on the quality of life and mental health of the 138 million women suffering from recurrent VVC (1–3).

The symptoms of VVC, which can include itching, burning, discharge, and dyspareunia, are caused by a hyperinflammatory response to *C. albicans* and not necessarily by the presence of fungus itself, which can commensally colonise the vagina. Therefore, understanding the mechanism by which *C. albicans* triggers inflammation could be key to preventing symptomatic infections.

Currently, guidelines for treating recurrent VVC involve six month maintenance therapy with azole antifungals, such as fluconazole. However, following discontinuation, over half of women suffer reinfections within the following six months (4). Moreover, there are reports of clinical isolates which are resistant to azole antifungals (5), underscoring the urgent need for novel treatment strategies.

Immunologically, host S100A8/A9 alarmin proteins have been implicated in vaginal inflammation during VVC, which is associated with non-protective neutrophil infiltration (6–8); however, no study has yet identified a fungal virulence factor responsible for immunopathology in women. Of note, S100A8/A9 (also known as calprotectin) is known to drive the expression of *C. albicans PRA1* (pH regulated antigen) (9), which encodes a secreted fungal protein with several immune-modulatory properties, including complement modulation and neutrophil chemotaxis (10–12). We have shown that the primary function of *C. albicans* Pra1 is to capture zinc from the environment and, as such, is regulated by this essential trace mineral (13); in fact the transcriptional induction of *PRA1* by calprotectin can be repressed with zinc (9).

Here we demonstrate that *C. albicans PRA1* is expressed in colonised and infected women and is required for inflammatory responses *ex vivo* as well as in mouse models of vaginal candidiasis. We show that zinc prevents inflammation in our experimental models and prevents reinfection in women who had been suffering from recurrent VVC.

## Results

### Expression of *PRA1* in women correlates with inflammatory cytokines

**Figure 1:**
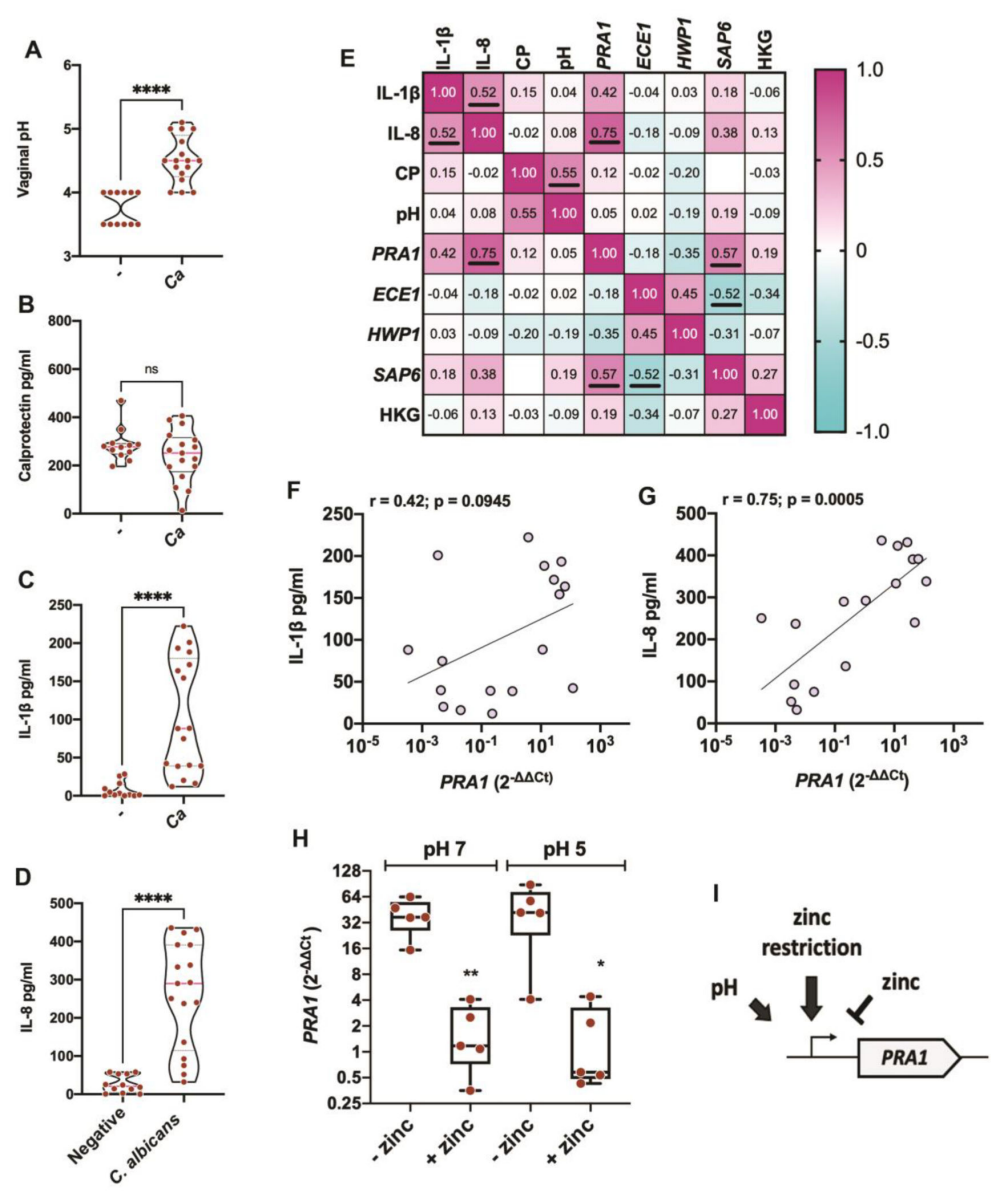
*PRA1* zinc scavenger gene expression correlates with inflammatory cytokine concentrations during *Candida albicans* vaginal colonisation and infection in humans. (**A** to **D**) Vaginal swabs from uninfected (n = 12) and *C. albicans* infected or colonised (n = 17) women were assessed for pH (A), calprotectin (B), IL-1β (C), and IL-8 (D). (**E**) Pearson correlation matrix of the *C. albicans*-infected samples showing the relationships between host parameters (displayed in **A-D**) and expression of the indicated fungal genes as determined by qRT-PCR (2^-ΔΔCt^); the mean RNA (pg/ml) of two housekeeping genes (HKG), *ACT1* and *CEF3*, was used as an indication of fungal burden. (**F** & **G**) Correlations between *PRA1* expression in women and cytokines IL-1β (F) and IL-8 (G) (r values from **E** and p values are displayed on graph). (**H**) *PRA1* expression in response to low zinc during vaginal epithelial tissue culture infection at both neutral and acidic pH. *C. albicans* (clinical isolate SC5314) was incubated on A-431 vaginal epithelial cells for 16h in RPMI at pH 7 or adjusted to pH 5, with or without 25 μM ZnSO_4_. Whiskers show minimum to maximum of all points. (**I**) Model showing the transcriptional control of *PRA1* in *C. albicans*. Statistical tests. **A-D**, Mann-Whitney test; **E**, Pearson r correlation displaying r values. Positive correlations are highlighted in magenta and negative in cyan. Significant (p < 0.05) r values are underlined in the matrix. **H**, t-test between vehicle and zinc treated samples. * p < 0.05; ** p < 0.01; **** p <0.0001.

Vaginal swab samples were collected from *C. albicans*-colonised and -infected (n = 17) and *C. albicans*-negative (n = 12) women and assessed for pH, calprotectin, inflammatory cytokines IL-1β and IL-8, *Candida* fungal burden, and expression of *PRA1*, as well as the well-established virulence genes *ECE1* (extent of cell elongation), *SAP6* (secreted aspartyl protease), and *HWP1* (hyphal wall protein) (table S1-S2). *C. albicans-colonised* and -infected women exhibited significantly higher pH than non-colonised individuals (P < 0.0001). Non-colonised pH ranged from 3.5 to 4.5 (mean pH 3.75) whereas *C. albicans*-colonised and -infected pH ranged from 4.2 to 5.1 (mean pH 4.57) (Fig. 1A). Calprotectin concentrations did not differ significantly between groups (P > 0.05), however a far narrower distribution was observed in uninfected individuals (Fig. 1B). In contrast, the proinflammatory cytokines IL-1β; and IL-8 were significantly higher in *C. albicans*-colonised and -infected samples than in negative controls (P < 0.001 in both cases, Fig. 1C-D).

We then excluded the negative samples from our dataset and performed a Pearson correlation analysis of all clinical parameters. *PRA1* was highly expressed in 7 out of 17 samples, likely as a result of local zinc restriction and the presence of host zinc-binding proteins. A positive relationship between the inflammatory cytokine IL-8 and *PRA1* expression was observed, whereas the relationship between *PRA1* and IL-1β was not significant (Fig. 1E-G). Of all the parameters measured, the strongest relationship observed was between *PRA1* and IL-8 (Fig. 1E-G).

Although *ECE1* was more highly expressed in some samples compared to *in vitro* yeast culture conditions, we did not observe a strong relationship between its transcript abundance and molecular markers of inflammation (Fig. 1E; Fig. S1).

We next sought to address a regulatory paradox: the human vagina is acidic and the *PRA1* gene (pH-regulated antigen 1) was originally named because it is expressed at neutral/alkaline pH (14). We have since demonstrated that the function of Pra1 is to scavenge zinc and, as such, the gene is also under tight regulatory control by the availability of this essential micronutrient (13).

To test whether *PRA1* expression can be zinc-regulated during vaginal infection, we developed an epithelial tissue culture model using the human vaginal epithelial cell line A-431. These cells have been used extensively to study *Candida* pathogenicity; however, commercially available culture media is neutral/alkaline. We selected a tissue culture media without zinc supplementation and then, to mimic the environment of the human vagina more closely, acidified it to pH 5.0. This had no obvious effect on the vaginal epithelia and, during a 16 h co-incubation with *C. albicans*, the pH did not change more than 0.8 units (Fig. S2). After infection of the vaginal epithelia in this acidified media, *PRA1* exhibited similar expression patterns as at pH 7. We reasoned that this was due to the low zinc content of the media. Indeed, media supplementation with 25 μM zinc sulphate fully repressed *PRA1* expression by *C. albicans* at both neutral and acidic pH (Fig. 1H). Therefore, although *PRA1* remains a pH-regulated gene (14), its regulation by zinc appears to dominate in this vaginal tissue culture model (Fig. 1I). Notably, we can effectively switch off *PRA1* expression by the addition of relatively low amounts of zinc.

### *Candida PRA1* is associated with neutrophil attraction

**Figure 2:**
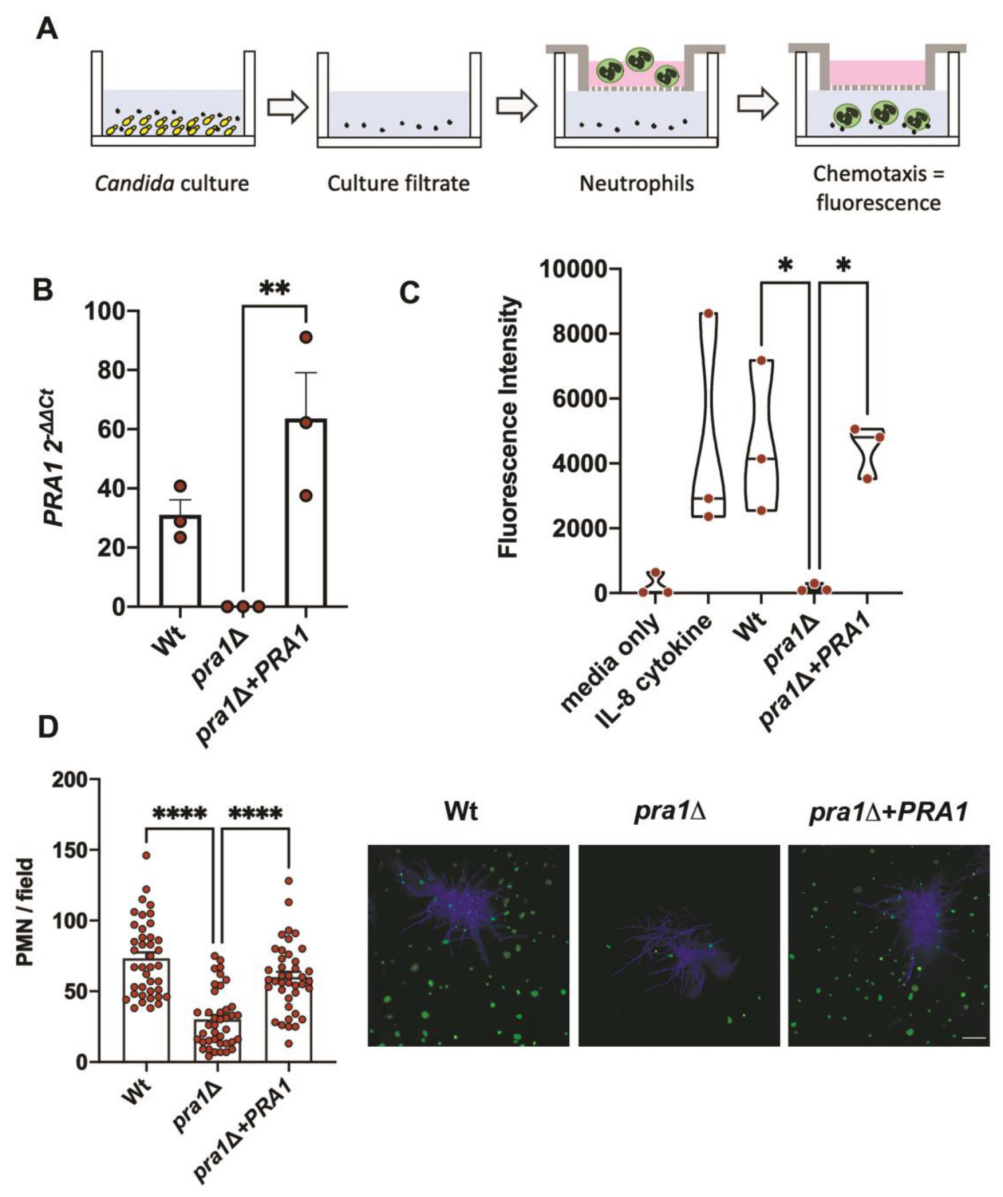
*PRA1* is essential for neutrophil attraction. (**A**) Schematic overview of the neutrophil chemotaxis assay. *Candida* culture filtrate was added to the lower compartment of the chemotaxis plate. Fluorescently labelled neutrophils were added to the upper compartment and chemotaxis assessed by measuring fluorescence in the lower compartment following migration of neutrophils through the membrane. **(B)** *C. albicans* wild type (Wt), *pralΔ* and *pra1Δ+PRA1* strains were incubated for 5 days in RPMI tissue culture media after which *PRA1* transcription was assessed by qRT-PCR (n = 3). **(C)** *PRA1* involvement in neutrophil chemotaxis. After the five-day incubation period, culture filtrate, unconditioned media (RPMI) or media containing neutrophil chemotactic factor IL-8 (100 ng/ml) were added to the lower compartment of the chemotaxis plate. Freshly isolated human neutrophils were fluorescently labelled with Calcein and added to the upper compartment. After 2 h incubation, neutrophil chemotaxis was determined by measuring the fluorescence intensity (at 485/530 nm) in the lower compartment (n=3). **(D)** As an alternative model, individual *C. albicans* yeast cells (~20 cells per well) were incubated in ibidi-VIII well microscopy slides in RPMI culture media for 10 h to induce microcolony development. Human neutrophils were added and incubated for a further 3 h. Samples were fixed and fungi stained with Calcofluor white (blue) and neutrophils with Sytox Green. Forty individual microcolonies per strain (from two independent experiments) were imaged and the number of neutrophils per field enumerated. Representative micrographs show the greater number of neutrophils in proximity to *PRA1*^+^ *C. albicans* microcolonies. Scale bar 150 μm. Statistical tests. **B-C**, one way ANOVA with Tukey post hoc test (excluding positive and negative control in **C**). **D**, Kruskal-Wallis test. Error bars show SEM. * p < 0.05; ** p < 0.01; *** p < 0.001; **** p <0.0001.

To further explore a role for Pra1 in vaginal inflammation, we employed an *ex vivo* human blood model of neutrophil chemotaxis (Fig 2A). All experiments used neutrophils isolated freshly from human blood; the gating strategy can be found in Fig S3. *C. albicans* wild type (Wt), *PRA1* deletion mutant (*pral*Δ) and genetic revertant (*pra1*Δ+*PRA1*) were incubated in RPMI tissue culture media for five days. Under these conditions all strains grew equally, and the pH remained neutral-alkaline (Fig S4). *PRA1* was expressed as determined by quantitative reverse transcription PCR (qRT-PCR; Fig. 2B) and Western blot (Fig S4). The culture filtrate from wild type *C. albicans* drove robust neutrophil recruitment comparable to a proinflammatory IL-8 positive control (Fig. 2C). Deletion of *PRA1* abrogated neutrophil chemotaxis, and genetic complementation of the *pra1*Δ mutant restored *PRA1* expression and neutrophil chemotaxis (Fig. 2B-C).

These data demonstrate that Pra1 is essential for neutrophil infiltration in the *ex vivo* human blood chemotaxis model. To complement this approach, we designed an alternative model for studying *C. albicans*—neutrophil interaction. Individual *C. albicans* cells were incubated on ibidi-VIII well microscopy slides in tissue culture medium for 10 h until they had formed hyphal microcolonies. Human neutrophils were then added, incubated for a further three hours, and the slides were washed and stained to visualise both fungi and immune cells. The number of neutrophils within the same field of 40 microcolonies per strain was enumerated. *PRA1*-expressing *C. albicans* microcolonies were associated with significantly higher numbers of neutrophils (Fig. 2D).

**Figure 3:**
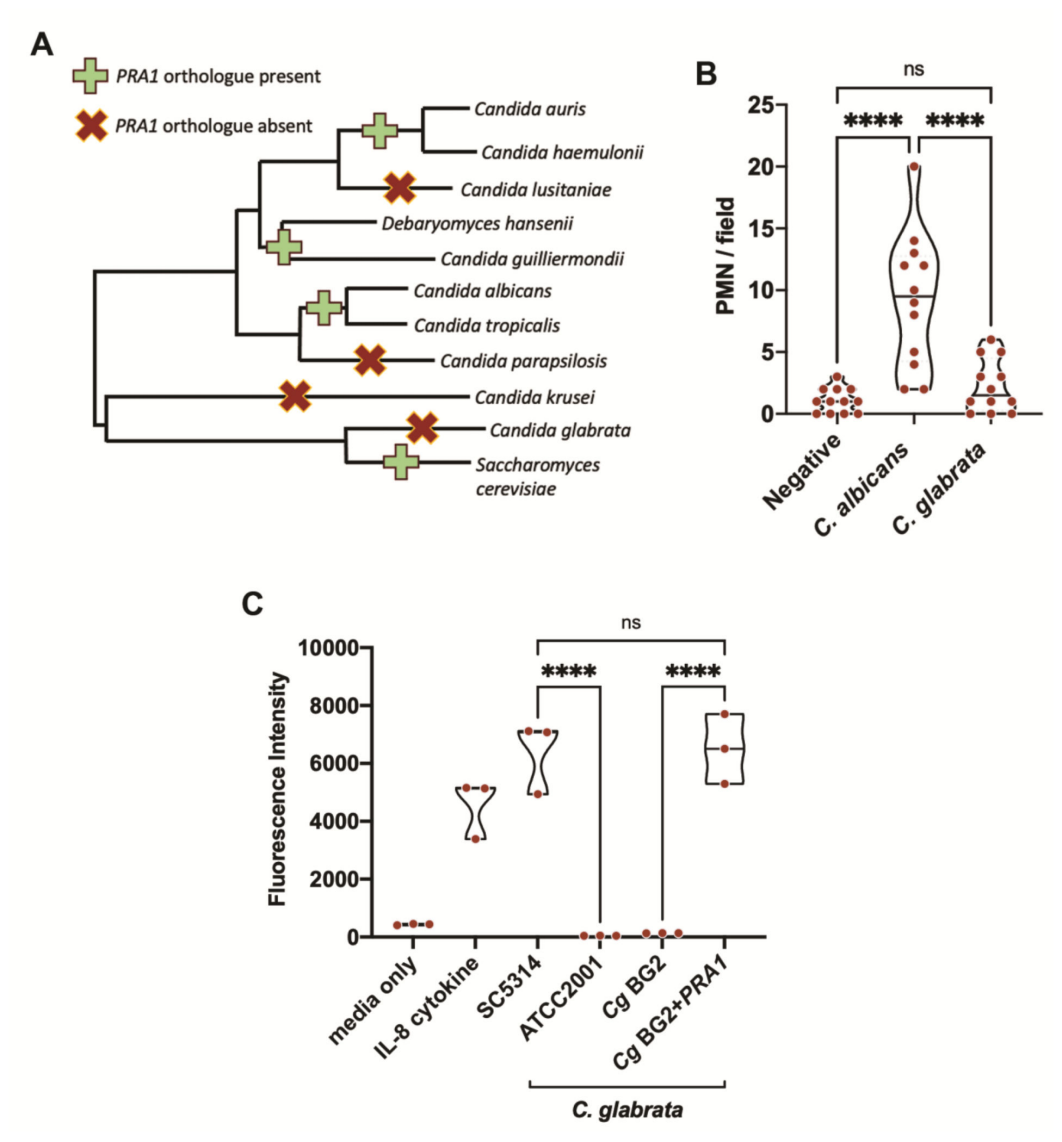
*Candida PRA1* genetic status influences neutrophil attraction. (**A**) The *PRA1* gene has been lost by certain *Candida* species including *C. glabrata*. A phylogenetic tree was drawn based on (Munoz 2018 Nat Comm) (17) and the presence or absence of *PRA1* orthologues determined by BLASTp. (**B**) Vaginal swabs from uninfected, *C. albicans*-infected, and *C. glabrata*-infected women were evaluated for neutrophil infiltrate. n=12 samples per group. (**C**) *PRA1* neutrophil chemotaxis assay. *C. albicans PRA1* from was codon-optimised for expression in *C. glabrata*, placed downstream of the *C. glabrata GPD2* promoter and transformed into *C. glabrata* BG2. Conditioned culture filtrate of *C. albicans* and *C. glabrata* strains with and without *PRA1* were used in a chemotaxis assay as outlined in Figure 2. Unconditioned media (RPMI), media containing neutrophil chemotactic factor IL-8 (100 ng/ml), or indicted culture filtrates were added to the lower compartment of the chemotaxis plate. Freshly isolated human neutrophils were fluorescently labelled with Calcein and added to the upper compartment. After 2 h incubation, neutrophil chemotaxis was determined by measuring fluorescence intensity (at 485/530 nm) in the lower compartment. n = 3. Statistical tests. **B** and **C**, One-way ANOVA with Tukey post hoc test. ** p < 0.01; *** p < 0.001; **** p < 0.0001.

We next examined the phylogenetic and genetic relationship between *Candida PRA1* and VVC immunopathology. The *PRA1* gene arose in the last common ancestor of the Dikarya (13) and is present in most extant fungal lineages (15). The Dikarya is the major sub-kingdom of fungi and encompasses ~98% of known species (16), including most pathogens, as well as industrially-relevant and food-producing species. However, certain species have lost the gene. This is true of *Candida glabrata*, which is the second most common cause of vaginal yeast infections (Fig. 3A, (13, 17)). *C. glabrata* vaginal infections are much rarer and their clinical pathology has not been reported.

Neutrophils from uninfected *C. albicans-* or *C. glabrata*-infected women (n=12 per group) were enumerated. The number of neutrophils per field ranged from zero to three in uninfected samples (mean 1.08); this was significantly higher in *C. albicans*-infected samples (P < 0.0001) ranging from two to 20, mean 9.25) (Fig. 3B). In contrast, *C. glabrata*-infected samples were associated with low numbers of neutrophils (ranging from zero to six, mean 2.25).

The association between *C. albicans* VVC and neutrophil infiltrate is in line with numerous previous studies (8). We are not aware of previous comparison between neutrophil numbers in *C. albicans* versus *C. glabrata* in clinical vaginal samples. Low neutrophil numbers in *C. glabrata*-infected women are consistent with observations made by one study that reported no signs of inflammation in a murine model of *C. glabrata* vaginal infection (18). It would therefore appear that vaginal *C. glabrata* infections in both mice and humans are associated with low neutrophil infiltrate.

To test if there is a functional relationship between these phylogenetic and clinical observations, we took a molecular-genetics approach. *C. albicans* and *C. glabrata* clinical isolates and laboratory strains were cultured in low zinc cell culture media as before and the filter-sterilised supernatant tested for chemotaxis in our *ex vivo* human neutrophil model. Again, *C. albicans* wild type strains’ culture filtrate promoted robust neutrophil recruitment comparable to that of the IL-8 positive control. In contrast, neutrophils did not migrate towards *C. glabrata* supernatant (Fig. 3C). We then placed a codon-optimised copy of *PRA1* from *C. albicans* under the control of the *C. glabrata GPD* promoter and introduced it into two independent *C. glabrata* genetic backgrounds; this resulted in the expression of *PRA1* in this otherwise *PRA1*-null species (Fig. S5A). The culture supernatants from these modified *C. glabrata* cells recruited neutrophils at levels comparable to that of *C. albicans* (Fig. 3C & Fig S5B). These data show that *PRA1* expression can “switch on” a neutrophil chemotactic response towards an otherwise unrecognised yeast.

### *PRA1* is required for vaginal inflammation *in vivo*

**Figure 4:**
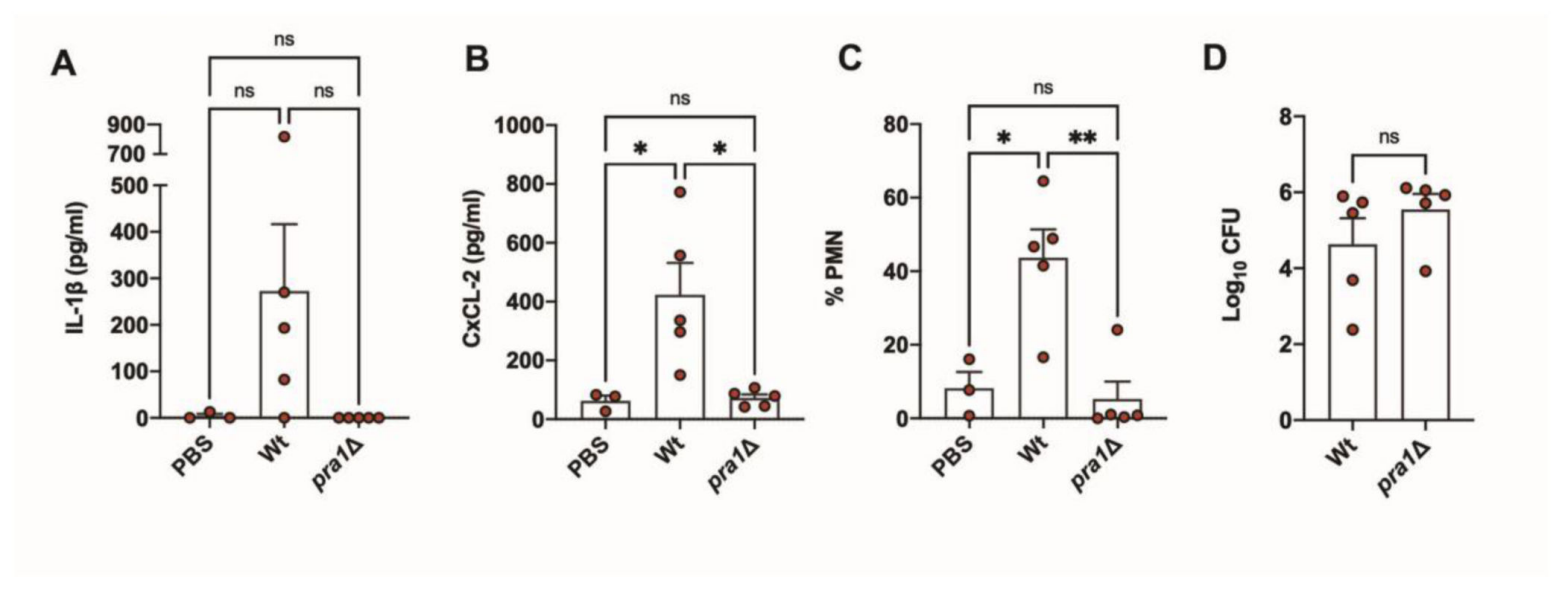
Deletion of *PRA1* prevents inflammation in a mouse model of vulvovaginal candidiasis. (**A-D**) Mice were vaginally infected with *C. albicans* wild type (Wt) or *PRA1*-deletion mutant (*pra1*Δ). 24 h post-infection, vaginal lavage fluid was collected and assessed for the presence of the proinflammatory cytokines IL-1β (**A**) and CxCL-2 (which has similarities to human IL-8; **B**) by ELISA. The presence of neutrophil (PMN) infiltration by flow cytometry (**C**) and fungal burden by plating colony forming units (CFU, **D**) were also assessed. Statistical tests. **A** to **C**, One way ANOVA with Tukey post hoc test. **D**, unpaired t-test. Error bars = SEM. * <0.05; ** p < 0.01 ns = not significant.

To test if Pra1 plays a functional role in vaginal inflammation *in vivo*, we used the well-established estradiol-treated mouse model of VVC. We used outbred CD-1 mice to try to better capture host genetic diversity. Vaginal infection with *C. albicans* resulted in a strong inflammatory response, including elevated concentrations of proinflammatory cytokines IL-1β and CxCL-2 (a murine cytokine with similarities to human IL-8) (Fig. 4A-B), as well as neutrophil infiltration (Fig. 4C, Fig S6). Notably, deletion of *PRA1* in *C. albicans* did not affect vaginal fungal burden as assessed by measuring colony forming units and wild type and *pral*Δ exhibited both yeast and hyphal morphologies (Fig. 4D, Fig. S7). However, deletion of *PRA1* blocked production of both inflammatory cytokines and neutrophil infiltrate (Fig. 4) but did not impact calprotectin levels (Fig. S8). Therefore, in the absence of *PRA1, C. albicans* is able to colonise the murine vagina without provoking inflammation.

### Zinc prevents immunopathology *in vivo*

Our observations suggest a connection between Pra1 and vaginal inflammation in the clinical setting as well as in our experimental infection models of VVC. Because the zinc-scavenging function of Pra1 is known (13), we reasoned that we may be able to prevent the symptoms of VVC with zinc supplementation. Of note, in humans, oral zinc supplementation does not affect zinc abundance in cervicovaginal lavage fluid (19).

**Fig. 5:**
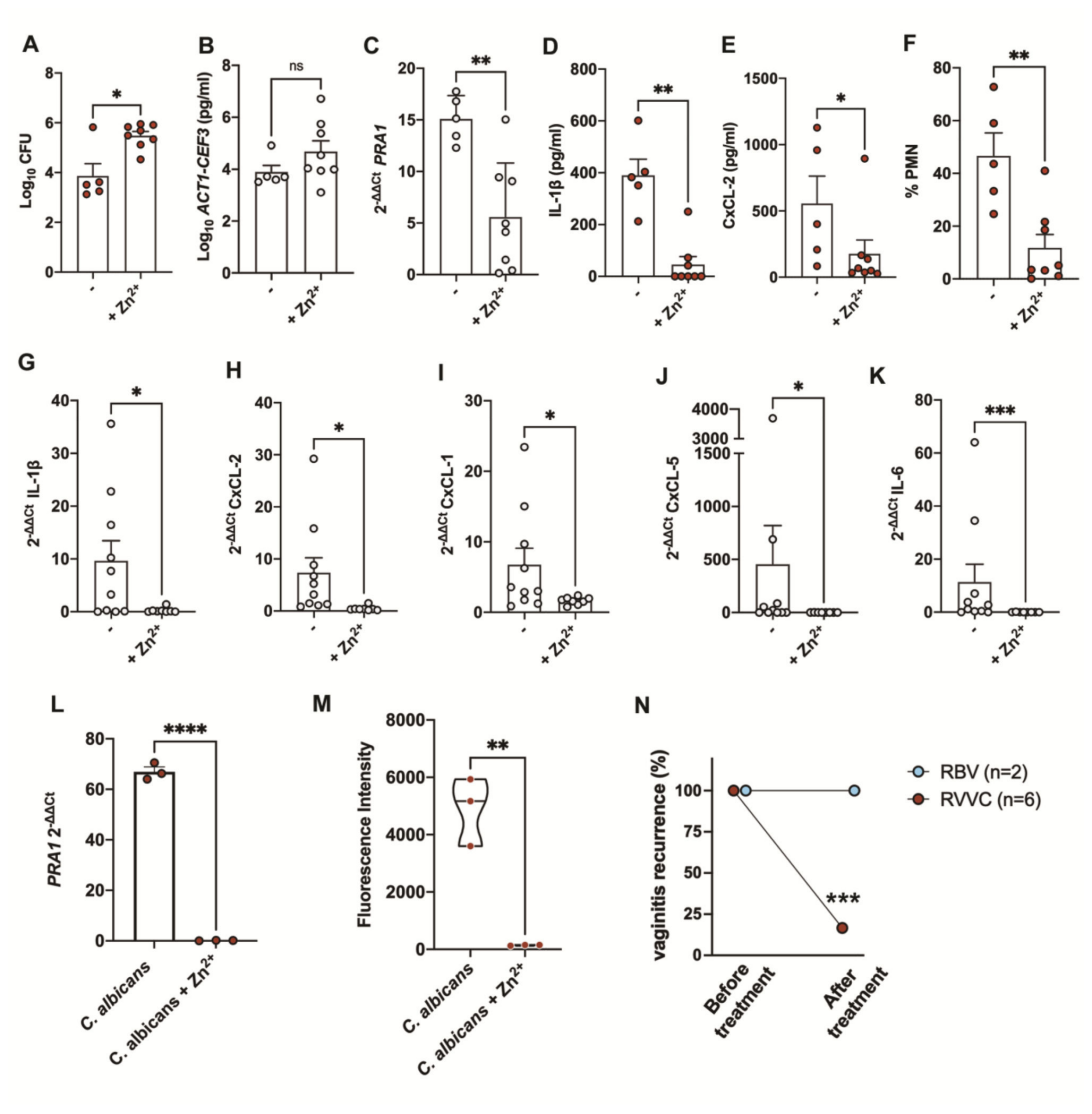
Zinc down-regulates *PRA1* and prevents vaginal candidiasis. Mice were vaginally infected with *C. albicans* wild type and administered with vehicle control or with zinc sulphate (+Zn^**2+**^, 10 μl at 25 μM) at the time of infection and 8 h post-infection. After 24 h, vaginal lavage fluid (red circles, **A, D-F**) or organs (grey circles, **B-C, G-K**) were collected. **(A to C)** Fungal burden was determined by measuring colony forming units (**A**) or expression of housekeeping genes *ACT1* and *CEF3* (**B**); *PRA1* expression was evaluated by qRT-PCR (**C**). (**D to F)** Luminal cytokines (IL-1β and CxCL-2, **D-E**) and neutrophils (PMN, **F**) were determined by ELISA and flow cytometry, respectively. (**G to K**) Vaginal tissue-associated expression of proinflammatory cytokines IL-1β (G), CxCL-2 (H), CxCL-1 (I), CxCL-5 (J), and IL-6 (K) were measured by qRT-PCR. (**L**) *C. albicans* clinical isolate SC5314 was incubated for five days in tissue culture medium with or without zinc supplementation and *PRA1* gene expression measured by qRT-PCR. (**M**) Culture filtrates were added to the lower well and calcein-labelled human neutrophils to the upper well of the chemotaxis system and neutrophil migration assessed by measuring fluorescence at 485/530 nm in the lower compartment after 2 h incubation. (**N**) In a retrospective study, eight women who had been suffering from recurrent vaginal infections used a zinc (20 μM) containing gel. Rates of reinfection for recurrent bacterial vaginitis (RBV n=2) and for recurrent vulvovaginal candidiasis (RVVC) were assessed following the three month study. Statistical tests. **A-K**, Mann-Whitney test. **L** and **M**, unpaired t-test; **N**, Paired t-test. Error bars = SEM. * p < 0.05; ** p < 0.005, *** p < 0.0005, **** p < 0.0001.

Therefore, using our mouse VVC infection model, we vaginally administered a zinc sulphate solution (10 μl at 25 μM) at point of infection and eight hours post-infection. We chose this concentration because it is non-toxic to both microbe and host (20, 21). Similar to *PRA1* genetic deletion, this micronutrient supplementation did not adversely affect *C. albicans* colonisation of the mouse vagina or fungal morphology (Fig. S7). In fact, zinc treatment increased the numbers of colony forming units recovered from mice (Fig. 5A). We have previously shown that zinc can reduce *C. albicans* adhesion to surfaces which may perturb fungal burden determination by the colony forming unit method (22) and therefore assessed fungal burden by measuring the expression of housekeeping genes *ACT1* and *CEF1*. This analysis revealed no significant difference in fungal colonisation in the control versus zinc-treated mice (P > 0.05; Fig. 5B). In contrast, zinc administration downregulated *C. albicans PRA1* expression *in vivo* by 2.6-fold (Fig. 5C). Zinc treatment of *C. albicans*-infected mice abrogated the production of both inflammatory cytokines (IL-1β and CxCL-2) (Fig. 5D-E) and reduced neutrophil infiltration to that observed in uninfected animals (Fig. 5F) without affecting calprotectin levels (Fig. S9). Gene expression of IL-1β, CxCL-2 (Fig. 5G-H) as well as CxCL-1, CxCL-5, and IL-6 (Fig. 5I-K) were all down-regulated in response to zinc treatment. Therefore, administration of relatively low amounts of zinc can down regulate *PRA1* in the mouse vagina and prevent inflammation, which is the hallmark pathology of VVC in humans.

To test whether zinc can prevent inflammation in humans we first returned to our *ex vivo* blood model. *C. albicans* was incubated for five days in tissue culture media with or without zinc supplementation. Zinc resulted in over 300-fold downregulation of the *PRA1* gene (Fig. 5L) and prevented chemotaxis of human neutrophils (Fig. 5M). Based on our molecular understanding of zinc acquisition by *C. albicans* and our observations on the link between *PRA1* and vaginal inflammation *in vitro, ex vivo, in vivo* and in the clinical setting, we performed a retrospective pilot study on the effect of zinc on vaginal infections in women.

Juvia is a moisturising gel which is used to treat vaginal dryness (20, 23). It contains 20 μM zinc sulphate heptahydrate, a concentration very similar to what we used in our experimental models of VVC. Full details of the study can be found in **the materials and methods**. We recruited ten women who were suffering from recurrent vaginal infections, defined as suffering from at least one infection every three months. The participants were asked to intra-vaginally self-apply 2 ml of the zinc-containing gel nightly for two weeks and then twice per week. Treatment failure was defined as reoccurrence within the first three months. Of the eight participants who completed the study, six had been suffering from recurrent VVC and two from recurrent bacterial vaginosis (RBV). The zinc gel had no effect on bacterial vaginosis (Fig. 5N). *PRA1* is unique to the fungal kingdom (13, 15), and our current model of vaginal inflammation does not encompass bacterial infections. In contrast, five out of six women (83%) who had previously been burdened with recurrent VVC did not suffer a reinfection within the three-month course of our study (Fig. 5N).

## Discussion

Our work has revealed an important intersection between innate and nutritional immunity. Zinc restriction (nutritional immunity) drives the expression of the fungal zinc scavenging protein Pra1. Although the zincophore plays an important role in micronutrient scavenging for the fungus, innate immunity has evolved to recognise and target this protein. Because the expression of nutrient scavenging proteins is a hardwired response to limitation of their substrate, this is a phenomenon wherein nutritional immunity creates an environment that induces expression of a neutrophil chemoattractant by the pathogen.

Inflammation is often critical for the resolution of fungal infections and the loss of *PRA1* by certain pathogenic species may represent an important step in their evolution. The *PRA1* gene emerged in the last common ancestor of the Dikarya (the major subkingdom of fungi) and, as such, is present in most sequenced fungi on the planet (13, 15). Therefore, our observations also raise important questions on the impact of *PRA1* locus status on the evolution of human fungal pathogens in general. Outwith the setting of VVC, neutrophils can play important roles in the control and killing of fungal pathogens. The loss of the *PRA1* gene by extant pathogenic species such as *C. glabrata* may therefore have had interesting effects on their interaction with our innate immune system in other disease settings. Given the evolutionary distance between *C. albicans* and *C. glabrata*, differences in *PRA1* locus status, and the notable differences in disease pathology observed in this study, we suggest that vaginal yeast infections caused by *C. albicans* and *C. glabrata* be considered fundamentally distinct diseases.

VVC is characterised by non-protective inflammation and our observations provide evidence that vaginal inflammation is driven at least in part by Pra1. Our study is limited by the small number of participants and a larger clinical trial would be needed to confirm these results; however, our findings could provide a simple means and a defined molecular mechanism to prevent vulvovaginal candidiasis. Because *C. albicans* Pra1 expression can be effectively dampened by low concentrations of zinc, this study suggests a potentially beneficial use for zinc in preventing the immunopathology associated with VVC. This could be particularly useful in cases of recurrent VVC which affects 138 million women and is currently very difficult to treat.

## Materials and Methods

### Study design

The objective of our study was to assess the contribution of the *Candida albicans* Pra1 protein to vaginal inflammation. For this we assessed clinical parameters of women colonised and infected with *Candida*, as well as the efficacy of a zinc-containing gel on preventing recurrence of clinical vaginal candidiasis. We also used genetically manipulated *Candida* strains, gene expression, as well as tissue culture, *ex vivo* and *in vivo* infection models to understand the mechanism by which *C. albicans* causes inflammation in vaginal tissue and how zinc treatment prevents this inflammation.

Each in vitro and ex-vivo experiment was performed in at least triplicate. A pilot study of mice was conducted to reduce the number of animals used, estimate variability among animals, and evaluate procedures and their effects. Mouse experiments were not blinded until sample collection. All in vitro and ex vivo experiments and sample measurements were blinded. The number of samples and the number of experimental replicates for each experiment are reported in figure captions. The minimum number of human samples collected was decided during the writing of the project and before ethical approval. All data were included in each figure, including outliers.

### Ethics statement

All methods were performed in accordance with relevant guidelines and regulations. For *ex vivo* chemotaxis with human neutrophils, blood samples were donated from healthy volunteers recruited from the MRC Centre for Medical Mycology at the University of Exeter (Exeter, UK). All participants provided written informed consent and donated a maximum of 100 mL of blood. This project was reviewed and approved by the University of Exeter CLES-Biosciences Research Ethics Committee (UECLES-Biosciences REC REFERENCE NUMBER: eCLESBio000371 V5).

All women recruited from a Hospital in the Umbria region (Italy) and from a Hospital in the Friuli Venezia Giulia region (Italy) signed informed consent in accordance with the Declaration of Helsinki. Local Ethical Committee CEAS (Comitato Etico delle Aziende Sanitarie, Umbria, Italy) approval was received for the Umbria region study (VAG1 n. 2652/15) on February 1, 2016. Local Ethical Committee CEUR (comitato etico unico regionale, Friuli Venezia Giulia, Italy) approval was received for the Friuli Venezia Giulia region study (RC 13/18-2715) on June 1,2018. The zinc gel study was approved by the University of Debrecen Regional and Institutional Committee of Science and Research Ethics (approval No. 5912-2021) and all enrolled patients signed informed consent.

CD1 female mice were provided from Charles River UK and maintained in the specific pathogen-free facilities of the University of Exeter. All animals were acclimatised for at least 1 week before starting experiments and were randomly assigned to experimental groups. Experiments were not blinded. All animal use was approved and in compliance with local University animal research ethical regulations and a UK Home Office project licence (P6A6F95B5). The physical condition of the animals was checked at least once daily until the end of each experiment.

### Vaginal human sample collection

The data reported in this study are from two different hospitals and were collected during two different periods. Group 1. The first group of women enrolled in this study attended a Hospital in the Umbria region (Italy) between 2016 to 2017. The group consisted of 36 non-pregnant, non-diabetic women, aged 21–40 years. These samples were used for neutrophil influx evaluation. 7 of them were also used to assess host and pathogen-related parameters, as detailed below. 12 women were negative for *Candida* isolation, 12 women were positive for *C. albicans* isolation, and 12 women were positive for *C. glabrata* isolation from a vaginal swab. Vaginal pH was measured directly from the vaginal swab by pH-Fix strips (Macherey-Nagel GmbH & Co.) and was in the range of 3.5-4.0 for non-colonised women and in the range of 4.0 - 5.0 for *Candida*-colonised women. No individuals presented any other vaginal co-infection (bacterial vaginosis or vaginitis). A vaginal swab was obtained from each participant, soaked in 1 ml of saline, and the presence of *Candida* was evaluated by plating the swabs on CHROMagar*Candida* (VWR InternationalPBI) and performing a MALDI-TOF test (Biomérieux S.A.). 100μL was examined under a light microscope (Olympus) to evaluate the presence of neutrophils (PMN) based on morphology. The number of polymorphonuclear PMN cells was counted in four fields at 40x magnification and expressed as average number of PMN/field.

Group 2. The second group of women enrolled at a Hospital in the Friuli Venezia Giulia region (Italy) consisted of 22 non-pregnant, non-diabetic women, aged 18–50 years, between June to September 2021 and used for different types of analysis. 10 women were negative for Candida isolation, 12 women were positive for *C. albicans* isolation and molecular identification.

At the enrolment, the women referred the eventual presence of symptoms (itching, burning, or abnormal discharge) and at gynecological examination clinical findings (abnormal vaginal discharge or erythema) of vulvovaginal infection were evaluated. Two vaginal swabs were obtained from each participant and soaked in 1 mL of saline. Immediately after, one sample was stained with hematoxylin and eosin to analyze the vaginal microbiota, neutrophil influx, Candida colonization, Candida morphology, presence or absence of lactobacilli, and presence or absence of any other types of bacteria. Using these parameters, a Nugent Score was assigned to each sample to evaluate the grade of vaginal dysmicrobism. The second swab was used to perform both Candida isolation on CHROMagar Candida (VWR International PBI) and DNA extraction for a multiplex PCR analysis to confirm Candida species (Alleplex Candidiasis Assay Seegene) and the presence of other bacteria responsible for vaginitis, vaginosis (Alleplex bacterial vaginosis/vaginitis assay, Seegen), or any other sexually transmitted infections (Neoplex 7 STI, Genematrix). All women involved in the current study had no co-infections other than *C. albicans*.

Vaginal swabs obtained from these 29 participants (7 from the first hospital in Umbria and 22 from the second one in Friuli Venezia Giulia) were centrifuge and the supernatant was used to quantify IL-1β, IL-8, and calprotectin concentrations as described in the supplementary materials. Cell pellets were used to quantify the expression of *PRA1, ECE1, SAP6* and *HWP1*. *ACT1* and *CEF3* were used as housekeeping gene and also to approximate the fungal burden for each woman. RNA extraction and gene expression were performed as reported in the method section above. Briefly, *Candida* RNA was extracted, treated with DNAse, quantified, and 500 ng RNA from each sample was converted to cDNA. Two different strategies were tried to evaluate *C. albicans* gene expression from the whole human vaginal swab. First, cDNA was immediately checked via RT-qPCR, but housekeeping genes were not immediately detected because most of the cDNA in each sample was from the host. For this reason, the cDNA was pre-amplified for 15 cycles in a normal PCR reaction using the AmpliTaq Gold DNA Polymerase kit (Thermo Scientific) before performing real time qPCR using the same amount of sample for all the genes tested. All *Candida* primers used are reported in table S4.

### Zinc-containing vaginal hydrogel pilot study

A retrospective cohort study was performed with the enrolment of 10 women with a history of recurrent vaginal infections at the outpatient clinic of the Department of Obstetrics and Gynecology, University of Debrecen, Hungary. Recurrent vaginal infection included recurrent bacterial vaginosis (RBV) and recurrent vulvovaginal candidiasis (RVVC). Enrolment criteria included the presence of RBV or RVVC and recurrent vaginal infection was defined as three or more vaginal infections in the last 12 months (ACOG Practice Bulletin). Exclusion criteria were a postmenopausal state, defined as individuals that had at least 12 consecutive months of amenorrhea without any other obvious reason or who had consistently elevated follicle-stimulating hormone blood concentrations of 30 mIU/mL or higher, local or systemic hormone therapy within the past six months, cytological atypia, prior radiation treatment, a history of breast, ovarian or other gynecological cancer, pelvic organ prolapse > stage 2, or recent use (3 months) of any vaginal product or douching. Women were asked to use a commercially available zinc-containing vaginal hydrogel (JUVIA vaginal gel; Fempharma, LLC.) as prophylactic treatment. The key gel ingredients are water, hydroxyethyl cellulose, 20 μM zinc sulphate, and lactic acid. The hydrogel was self-applied intra-vaginally via a vaginal applicator. Participants were asked to place 2 ml of gel into the vagina nightly for two consecutive weeks and, after that, twice per week. Women were asked to return to the clinic if any symptoms of vaginal infection were present for evaluation. Patients underwent a detailed gynaecological exam at enrolment and the follow-up visit. At the first visit, general gynaecological and medical history was taken, including age, body mass index (BMI), previous pregnancies, deliveries or operations, menstruation cycle, hormonal therapy, current relationship status, sexual partners in the last 12 months, episodes of vaginitis over the previous 12 months. Demographic and pertinent clinical information was recorded prospectively and stored in a dedicated database.

### Statistical analysis

Before performing statistical analyses, GraphPad Prism 9.0 software was used to test the normal distribution of each data set. Based on the distribution (normal or not normally distributed) the appropriate statistical test was chosen and performed as described in each Figure Legend. GraphPad Prism 9.0 software was used to perform most statistical analyses apart from the zinc gel study in Figure 5N, where Microsoft Excel 2019 was used. Statistical tests and P values are described in the manuscript and Figure Legends. P < 0.05 was considered significant.

## Supplementary Material

Supplementary Material

## Data Availability

All data are available in the main text or the supplementary materials. Raw data from Figures is given in data file S1.

## One Sentence Summary

Application of the trace mineral zinc in a gel form prevents symptoms in women with recurrent vaginal yeast infections.
