# Supplementary material for: Zinc prevents vaginal candidiasis by inhibiting expression of an inflammatory fungal protein
Source: Sci Transl Med. Author manuscript; Available in PMC 2024 Jun 6. (PMC7616067; doi:10.1126/scitranslmed.adi3363)
Supplement: Supplementary Material [file EMS192721-supplement-Supplementary_Material.pdf]

Materials and Methods

Cell line and *Candida* strains

Tissue culture experiments were carried out using the A-431 vaginal epithelial cell line obtained from ATCC and maintained in Dulbecco's Modified Eagle's Medium (DMEM, Gibco) supplemented with 10% heat-inactivated foetal bovine serum (FBS, Gibco) (DMEM complete medium) at 37°C + 5% CO_2. Human vaginal epithelium multilayer (VEm) was obtained by the differentiation of A-431 cells ($1x10^6$/ml) in a 24 well-plate in complete medium. Prior to stimulation and infection, the VEm was incubated in serum-free DMEM for 2h, and all experiments were carried out in serum-free DMEM at pH 5 or pH 7 (± 0.2). The acidic pH was obtained using 0.1 N HCl in all the experiments. In preliminary experiments, media was also acidified using DL-lactic acid (Sigma) to evaluate the effect on the epithelium from different molecule, but no difference was found.
The *Candida* strains used in this study are listed in table S3. Strains are available to other researchers upon request. For experiments examining the impact of *PRA1* deletion on *C. albicans*, the isogenic wild type (referred to as Wt in figures) BWP17+CIp30 (Mayer, 2012, PLoS Pathogens) was used together with *PRA1* null (*pra1*Δ) and genetic revertant (*pra1*Δ+*PRA1*) (Citiulo, 2012, PLoS Pathogens). For experiments examining the impact of zinc treatment on *PRA1* expression, the clinical isolate SC5314 was used.
Candida glabrata harbouring *PRA1* was created as follows. DNA encoding the *C. albicans* Pra1 (*Candida* genome database Arnaud, 2005 Nucleic Acid Res) was codon optimised for expression in *C. glabrata* (https://eu.idtdna.com/CodonOpt) and synthetically generated at Eurofins with attp1 tails adaption added to ensure compatibility with the Gateway cloning system. The *C. glabrata*-optimised *PRA1* gene was cloned into the pDONR221 entry vector using GATEWAY cloning techniques and then shuttled into pAG423GPD-ccdB destination vector (Addgene) using the LR clonase reaction.
Destination vectors were transformed into *C. glabrata* via electroporation (24). The *C. glabrata* parental strain were histidine auxotrophs of BG-2 (Cormack, 31, Genetics) and CBS138 (Kitada, 1995, Gene; Dujon, 2004, Nature). Transformants were selected for growth on SC-HIS media and four independent transformants from genetic backgrounds were used in experiments. The presence of *PRA1* transcript in these strains was confirmed using quantitative reverse transcription polymerase chain reaction (qRT-PCR) with primers CgPRA1-for (GGCCGACTGGGTTAAAGGTT) and CgPRA1-rev (ACCTCAGCACTTGCAGTCTC).
C. albicans and *C. glabrata* cultures were maintained on YPD agar medium (1% yeast extract, 2% peptone, 2% dextrose, 2% agar). For primary precultures, SD (synthetic defined) minimal medium (1X YNB without amino acids, Difco, 2% glucose) was used. Liquid cultures were incubated with shaking (200 rpm) at 30°C for 24 h. Cells were washed twice with 1 mM EDTA and then twice with double distilled milliQwater. These were used to inoculate SD without zinc (YNB without zinc and amino acids, Formedium, 2% glucose), RPMI 1640 medium without phenol red (Gibco) or (for A-431 tissue culture) DMEM (above)
Candida cells were always washed in sterile phosphate buffered saline (PBS) (Gibco) and adjusted to the required cell density before each experiment.
All cell types (*Candida,* mouse, described below, and human cell line), were counted using two different methods: the Vi-cell Blu cell viability analyser (Beckman Coulter, Life Sciences) and the Hemocytometer after dilution in trypan blue.

In vitro vaginal epithelium infection

C. albicans SC5314 strain was pre-cultured in SD minimal media, washed as described above and inoculated into SD without zinc to OD_{600} 0.1. Both cultures were for 24 h at 30°C and 200 rpm. Human vaginal epithelium (A-431) in 24 well plate was infected with 5×10^4 *C. albicans* / well for 16h in the presence or absence of 25 µM zinc sulphate (Sigma), in DMEM pH 7 or pH 5. After 16 h, pipette tips were used to gently scrape the bottom of the well to recover the cells. The recovered cells were pelleted and used immediately for the RNA extraction to evaluate *C. albicans* gene expressions. The pH of the cultures was measured before and after incubation using a pH meter (Jenway 3520).

RNA extraction and gene expression quantification

Candida and/or mouse RNA were extracted following the instructions of RiboPure - Yeast Kit. After DNAsi treatment, RNA integrity and concentration was confirmed using a nanodrop spectrophotometer and 500ng RNA from each sample was reverse transcribed to cDNA using RevertAid first strand cDNA synthesis kit and following the kit instructions. To evaluate gene expression a real time qPCR (Quant Studio 7 pro, Applied Biosystem, Thermo Fisher Scientific) was performed using Syber green (Power up sybr green master mix). For real-time PCR reaction,

1 µl - 500 ng of cDNA was used for each sample and each gene. All the *Candida* and mouse primers used are reported respectively in Table S4 and S5. All primers were designed using SnapGene software and showed similar capacity and efficiency in detecting expression of the evaluated genes. Before being used, all primers were checked and analyzed with Oligo Analyzer 3.1 IDT and BLAST against all available *C. albicans* genome sequences to ensure that they don't align to hypervariable gene regions. For determining gene expression in *C. albicans*, genes of interest were normalised with two housekeeping genes (*ACT1* and *CEF1*), except for the effect of zinc treatment on *PRA1* expression *in vitro*, where *PRA1* was normalised by *ACT1* only. To detect the presence of *PRA1* transcript in *C. glabrata* + *PRA1* strains, expression was normalised by *ACT1*. Genes expression levels were calculated by the comparative Ct method ($2^{-\Delta\Delta Ct}$ formula) after normalization with the average housekeeping genes and with an *in vitro / vivo* inoculum. To approximate *C. albicans* fungal burden in human and mice samples, the quantity of RNA per µl for each sample was calculated using a standard curve made with the *C. albicans in vitro / vivo* inoculum. Amplification conditions were the same for all the genes examined.

Protein expression

To assess Pra1 protein in culture supernatant, *C. albicans* cells from an SD minimal media preculture (30°C, 200 rpm) were inoculated into RPMI without phenol red (3 × 4 ml, universal flask) and incubated with 150 rpm shaking at 24°C for a further six days.

The cultures were then pooled, pelleted (4,000 rpm, 10 min), the supernatant filter sterilised and protein precipitated using trichloroacetic acid. 10 µl of concentrated protein were run on a 4-20% polyacrylamide gel, and stained for 25min with Coomassie dye. The gel was then washed three times for 10min in 50% EtOH and 40% acetic acid and washed overnight in water. The proteins were transferred to a membrane which was then blocked with 5% BSA in TBS-T for 2 h. The membrane was incubated with a primary rabbit Anti-Pra1_albicans polyclonal antibody (Thermofisher) diluted 1:5000 in TBS-T, washed three times for 10 minutes each in TBS-T before addition of the secondary goat Anti-rabbit IgG-HRP (Cell Signaling) diluted 1:3000. The membrane was then washed three times with TBS-T and imaged on a ChemiDoc MP Imaging System (Bio Rad).

Damage assay and epithelium integrity checking

The integrity of A-431 tissue culture human vaginal epithelia were evaluated after incubation with acidic DMEM microscopically using EVOS M5000 and Olympus EP50/CKX3-SLP microscope at 10x magnification. Epithelial cell damage was determined by the release of LDH into the culture media using a Cytotoxicity Detection kit (LDH, Invitrogen). The unmodified (pH 7) tissue culture media was always used as a control, plus all the negative and positive controls provided in the kit. The percentage cytotoxicity of epithelial cells was calculated as follows: sample of interest LDH release minus means background cells LDH release / means maximal LDH release minus means background cells LDH release per 100.

Isolation and preparation of neutrophils

Human peripheral blood neutrophils (PMN), obtained from healthy volunteers, were separated by density gradient centrifugation on FicollPaque Plus, followed by the hypotonic lysis of erythrocytes using a 1X red blood cells lysis buffer (10X recipe: NH_4Cl 1.55 M, $NaHCO_3$ 120 mM, EDTA 1mM, in sterile ddH_2O) using the protocol established by Chang Cui et al (26). Neutrophils were then washed twice with PBS and resuspended in 5 ml RPMI-1640 without phenol red containing 10% heat-inactivated FBS. The cells were then incubated with Calcein AM, fluorescent cell permeable derivative of calcein (Sigma Aldrich) (5 µg / ml) for 30 min at 37˚C + 5 % CO_2. After the incubation, the neutrophils were washed twice with PBS, counted, and resuspended in the same complete medium at 5 x 10^6 / ml.

***Candida* supernatant preparation for *ex-vivo* neutrophils chemotaxis test.**

To test neutrophil chemotaxis, *Candida* supernatant was prepared in the following ways. *Candida* strains were cultured for 24 h in SD minimal media at 30°C, 200 rpm, washed twice in milliQwater, and resuspended in RPMI without phenol red at 1 x10^6 cells/ml.

The *C. albicans* strains were distributed in triplicate in a 24-well plate, 1 mL per well, and incubated at 24˚C for 5 days, 130 rpm shaking. In selected experiments only *C. albicans* clinical isolate SC5314 was cultured in the presence or absence of 25 µM of zinc sulphate. One extra well for each strain and for each condition was prepared to measure the pH value over the time using a pH meter (Jenway 3520) and pH indicator paper test strips (pH 0.0 to 14.0).

The supernatant obtained for the *C. glabrata* experiment were generated as described above but incubated 30˚C for 5 days, 100 rpm shaking.
In Fig. S4 the variation on the pH value over the time for one *C. albicans* strain is showed. Importantly, under these culture conditions, all three *C. albicans* strains grew the same (Fig. S4). In both cases, supernatant was collected, and filter sterilized (0.2 µm filter, (Fisher) and the cells were frozen at -80˚C for RNA extraction.

Ex-vivo human neutrophil chemotaxis.
Human neutrophil migration was measured using a 96-well chemotaxis chamber with 3.2 mm diameter filter with membrane porosity of 5 µm (Neuro Probe). Calcein AM fluorescently labelled human neutrophils (5 x 10^6/ml) were transferred into ChemoTx filters placed in the 96-well plates containing 300 µl of either medium alone (RPMI without phenol red, negative control), media containing recombinant IL-8 (100 ng/ml) (Biochem, positive control) or *Candida* culture filtrate supernatant. The chamber was incubated for 2 h at 37°C + 5% CO_2. Following incubation, the non-migrating cells on the top of the filter were removed by gently wiping the filter and the cells that had migrated into the bottom chamber were measured by fluorescence signal (excitation, 485 nm; emission, 530 nm) using a Tecan Spark plate reader.

Ex vivo human neutrophil-*Candida* microcolony proximity assay.
C. albicans strains were precultured in SD minimal medium and then in SD 0, for 24 h, 30˚C, with 200 rpm shaking. Cells were washed twice in milliQwater, approximately 20 cells were added to 250 µL of RPMI without phenol red pH in 8-well IbiTreat Slide (Ibidi) and incubated for 10 h at 37˚C + 5% CO_2. 2 x 10^4 human neutrophils isolated as described above, were added to each well and incubated for an additional 3h. Wells were washed, which removed non-adherent neutrophils, stained with Calcofluor white (1 µg / mL) for *Candida* and SYTOX green nucleic acid stain (2.5 µM Invitrogen) for neutrophils All the staining was performed on ice for 20 min. The cells were washed 3 times with PBS and then fixed with 1% paraformaldehyde for 10 min on ice. Each well was then evaluated using a DeltaVision Microscope (ImSol Image Solution) at 20x magnification. For each *Candida* microcolony the number of neutrophils per field were counted. The fluorescent image overlays were generated using ImageJ. The results are expressed as number of neutrophils for each field including one *Candida* microcolony.

Neutrophils characterization with flow cytometer.
Human neutrophil isolated from healthy volunteers were checked by flow cytometer. Neutrophils were counted and adjusted at 1 x 10^6 / tube. The cells were stained with a viability dye (fixable Viability Dye-eFluor450, from Thermo Fisher) in PBS for 20 min on ice in the dark.
Following viability staining, cells were washed and resuspended in flow staining buffer (PBS + 10% FBS), for 15 min on ice in the dark, to block unspecific binding. For cell surface staining, cells were then washed and recovered by centrifugation and 100 µL of antibody cocktail in flow staining buffer were added and incubated for 30 min on ice in the dark. The cells were then washed with staining buffer, fixed with 1.5% paraformaldehyde and analysed. In a preliminary test, the same cells were fixed or not fixed to evaluate the antibody staining in the presence or absence of fixation. No difference was found.
The following antibodies were used: Alexa Fluor 700 CD2 (RPA-2.10, 0.20 µg/test), PE-Cyanine7 CD14 (61D3, 0.5µg/test), APC CD15 (MMA, 0.125 µg/test), PE CD16 (eBioCB16(CB16) 0.1µg/test), FITC CD45 (HI30, 0.20 µg/test) and APC-eFluor 780 CD56 (CMSSB, 0.10 µg/test). All antibodies were from eBioscience (Thermo Fisher). Control samples including unstained, isotype controls (all from eBioscience, Thermo Fisher), single stain (UltraComp eBeads Compensation Beads, Thermo Fisher), heat-killed cells with viability dye, and Fluorescence Minus One (FMO) were acquired to determine the boundary and specificity of the fluorescence signal. The same number of events were acquired for each sample (10,000). Flow cytometry was performed on Attune NxT Flow Cytometer (ThermoFisher Scientific) and analysed with FlowJo 10 software (BD Biosciences). Gating strategies used are shown in Fig S3.

Neutrophils characterization with Wright-Giemsa stain.
The purity of human neutrophils was also checked using a Wright-Giemsa Stain (abcam) following the kit instruction. The slides were prepared using a Cytospin 4 centriguge (Thermo Scientific) and then evaluated microscopically using EVOS M5000 at 40x magnification.

Vaginal mouse infection model
Female CD1 mice obtained from Charles River (UK) were used at 6-8 weeks of age, 18-20 g of weight. Mice were allowed to acclimatise for 1 week before starting the experiment; after this period, mice were weighted daily until

the end of the study. In this mouse model the mice did not show any sign and symptoms of distress and they did not show a decrease in the body weight over the time.
Mice were maintained under a pseudoestrus condition by subcutaneous (s.c.) injection of 0.2 mg of estradiol valerate in 100 µl of sesame oil (both from Sigma-Aldrich) 3 days before the infection.
C. albicans strains were first pre-culture in SD minimal media for 24h at 30˚C, 200 rpm shaking, then washed twice as described above and cultured in SD 0 limited media for five days at 30˚C, 200 rpm shaking. Over the 5 days, the SD 0 limited media was replaced twice.
The day of infection (day 0) mice were vaginally inoculated with 10 µl of *C. albicans* cell suspension containing 2 x 10^7 fungal cells in PBS or with PBS alone.
To allow vaginal contact and adsorption of the inoculum, mice were held head down for 1 min following inoculation. In selected experiments mice were also treated or not intravaginally with 25 µM / 10µL of zinc sulphate, immediately and after 8 h of infection.
At day 1 post-infection, the vaginal lumens were thoroughly washed with 300 µl of PBS, given in six separate 50 µl volumes. The vagina was also surgically collected.

Mouse vaginal lavage: flow cytometry, colony-forming units assay and cytokines quantifications
Cells in the vaginal lavages were counted using Vi-cell Blu cell viability analyser. Before centrifugation, fifty microliters of vaginal fluid were used to perform 10-fold serial dilutions. 100 µL of each dilution were plated in Sabouraud dextrose agar with chloramphenicol (50 µg / mL) (SAB + C) plate in duplicate and incubated at 30°C for 48 h. Fungal load was expressed as the Log_{10} number of colony-forming units (CFU) in 300 µl of PBS (total vaginal washes). For microscopy, *C. albicans* cells were stained with Calcofluor white (1 µg / mL).
The rest of the vaginal washes were centrifuged, and the supernatants were used for cytokine quantification (IL-1β and CxCL-2) as described below in the cytokine's quantification section.
The cells were stained for flow cytometry using the standard methodology described above in the previous section to quantify the local PMN recruitment.
The following antibodies were used: FITC CD45 (30-F11, 0.20 µg/test), PE Ly-6G (1A8-Ly6g, 0.20 µg/test) and APC CD11b (M1/70, 0.20 µg/test). In selected experiments also APC-eFluor™ 780 CD326 (EpCAM) (G8.8, 0.20 µg/test) was used to analyse the epithelial cells population. All antibodies were from eBioscience (Thermo Fisher). Control samples including unstained, isotype controls (all from eBioscience, Thermo Fisher), single stain (UltraCompTMeBeads Compensation Beads, Thermo Fisher) and Fluorescence Minus One (FMO) were acquired to find out the boundary and specificity of the fluorescence signal. All the cells were acquired for each sample (total volume 300 µL). Flow cytometry was performed on Attune NxT Flow Cytometer (Thermo Fisher Scientific) and analyzed with FlowJo 10 software (BD Biosciences, USA). Gating strategies used are shown in Fig. S6. Neutrophils recruitment is expressed as the percentage of neutrophils in the total cells recovered from the total vaginal wash from each mouse. Of the entire $CD45^+$ population in *C. albicans* wild type-infected murine vaginas, over 90% were $CD11b^+Ly\text{-}6G^+$ neutrophils. Therefore, other immune cell populations were not evaluated in this study.

Mice vagina processing: colony-forming units assay and mouse/*Candida* gene expression
All organs were weighed immediately after harvest and then homogenised (Homogeniser IKA T25 easy clean digital). The samples were then filtered using sterile cell strainer 100 µM (Nylon Mesh) and 50 µL of vaginal homogenate or 100 µl of 10-fold serial dilutions were plated in SAB + C plate in duplicate and incubated at 30°C for 48 h. The fungal load was expressed as the Log_{10} number of colony-forming units (CFU) per gram of tissue. The CFU number from vaginal lavage and from the organ was comparable for each individual mouse, demonstrating that either CFU method (lavage or tissue) can be used to evaluate fungal burden in this infection model.
The samples were then centrifuged, and the pellet was frozen for RNA extraction following the method described above. Briefly, *Candida* and mouse RNA were extracted, treated with DNAsi treatment, quantified and 500 ng RNA from each sample was reverted in cDNA. To evaluate mouse gene expression a real time qPCR was performed with 1 µl-500 ng of cDNA using Syber green reaction. For the *C. albicans* gene expression from the whole mouse vagina, two different strategies were tried. First, the cDNA was immediately checked in a real time qPCR reaction, but the housekeeping genes were not able to be immediately detected given that the most of the cDNA in each sample was from the host. For this reason, the cDNA was then pre-amplified for 15 cycles in a normal PCR reaction using the AmpliTaq Gold DNA Polymerase kit (Thermo Scientific) before performed the real time qPCR using the same amount of sample for all the genes tested. This pre-amplification cycle was not necessary for the *in vitro* experiments or for the mouse genes. All the *Candida* and mouse primers used are reported respectively in Table S4 and S5. Two housekeeping gene were used each time and the average value of each sample was used for the calculations. Two types of analysis were performed based on the different experiments as reported in the "RNA

extraction and gene expression quantification" section. The genes evaluated for the *C. albicans* were *ACT1*, *CEF3,* and *PRA1* and for the mouse *GAPDH*, *IL-1β, CxCL-2, CxCL-1*, *IL-6*, *CxCL-5*.

Cytokine production and quantification

The supernatants from vaginal samples from mice (lavage) and humans (swab) were collected and tested for human or mouse IL-1β (Invitrogen), human IL-8 (Invitrogen), human Calprotectin (CalproLab), mouse CxCL-2 (MIP-2) (R&D Systems), mouse S100A8 (MyBiosource) by specific ELISA assay. Cytokine titre was calculated relative to standard curves.

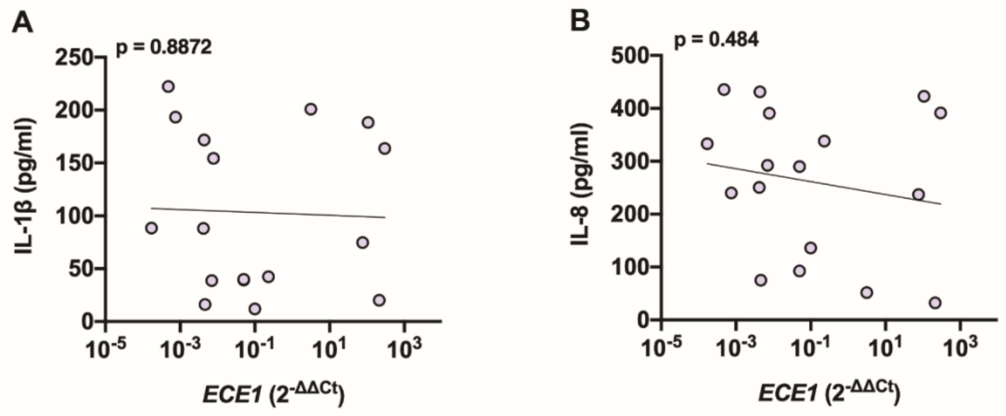

Fig. S1.

Expression of the *ECE1* gene does not correlate with inflammatory cytokine concentrations in clinical samples. *ECE1* transcript was determined by quantitative reverse transcription PCR and IL-1β (**A)** and IL-8 (**B**) by ELISA during *C. albicans* vaginal colonization and infection in human vaginal samples. There was no significant correlation between *ECE1* and either inflammatory cytokine.
Statistical test: Pearson r correlation. The r values are reported in Figure 1 **E**.

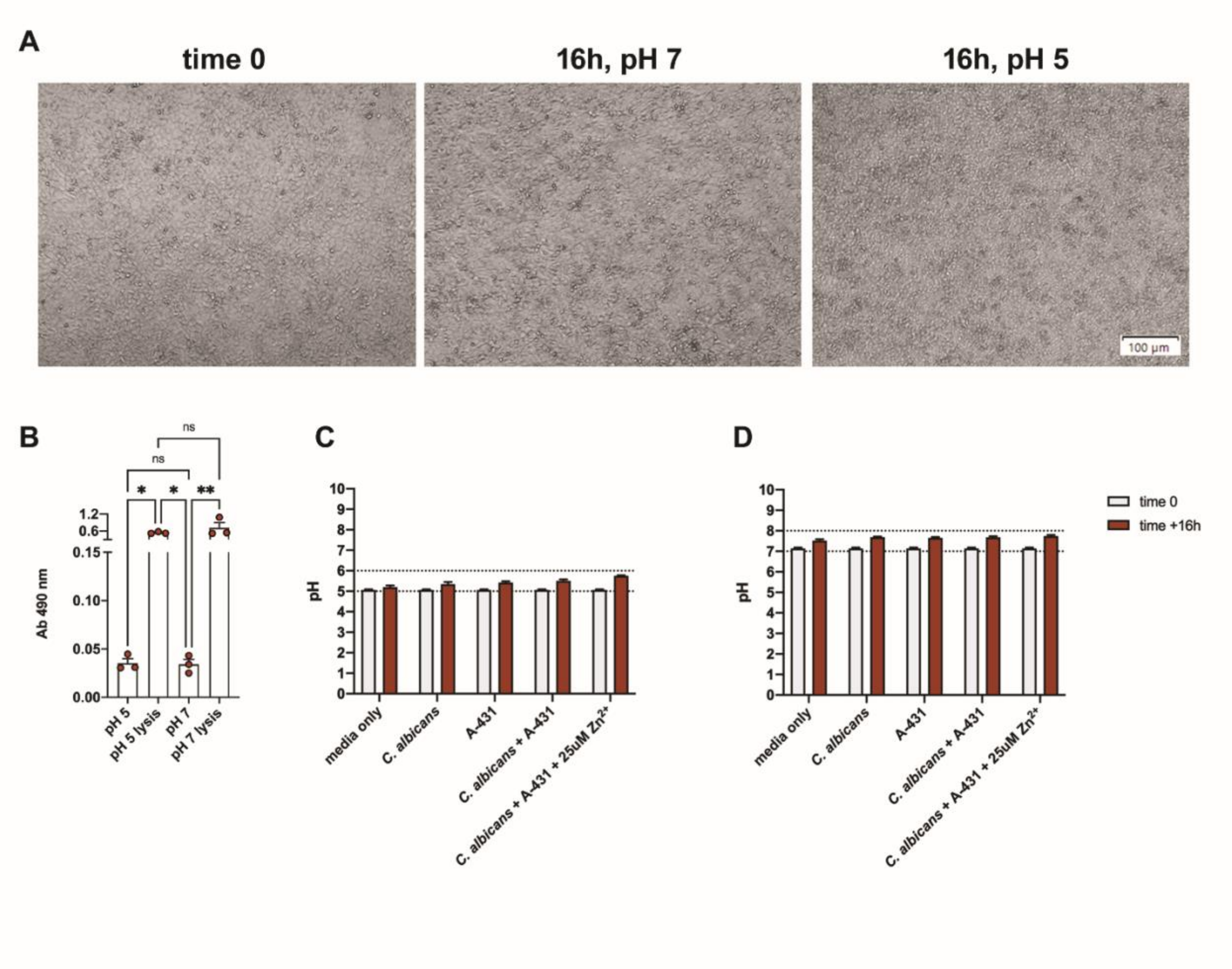

Fig. S2.

Integrity and damage evaluation of epithelial tissue culture model at acidic and neutral pH. The integrity of A-431 tissue culture human vaginal epithelia were evaluated microscopically after incubation with acidic pH 5.0 and standard pH 7.0 DMEM following 16 h incubation (**A**). Epithelial cell damage was determined by the release of LDH into the same culture media (**B**). During the 16 h co incubation between epithelium and *C. albicans*, the pH was also measured at pH 5 (**C**) and at pH 7 (**D**).

Statistical test: unpaired t-test. * <0.05; ** <0.01

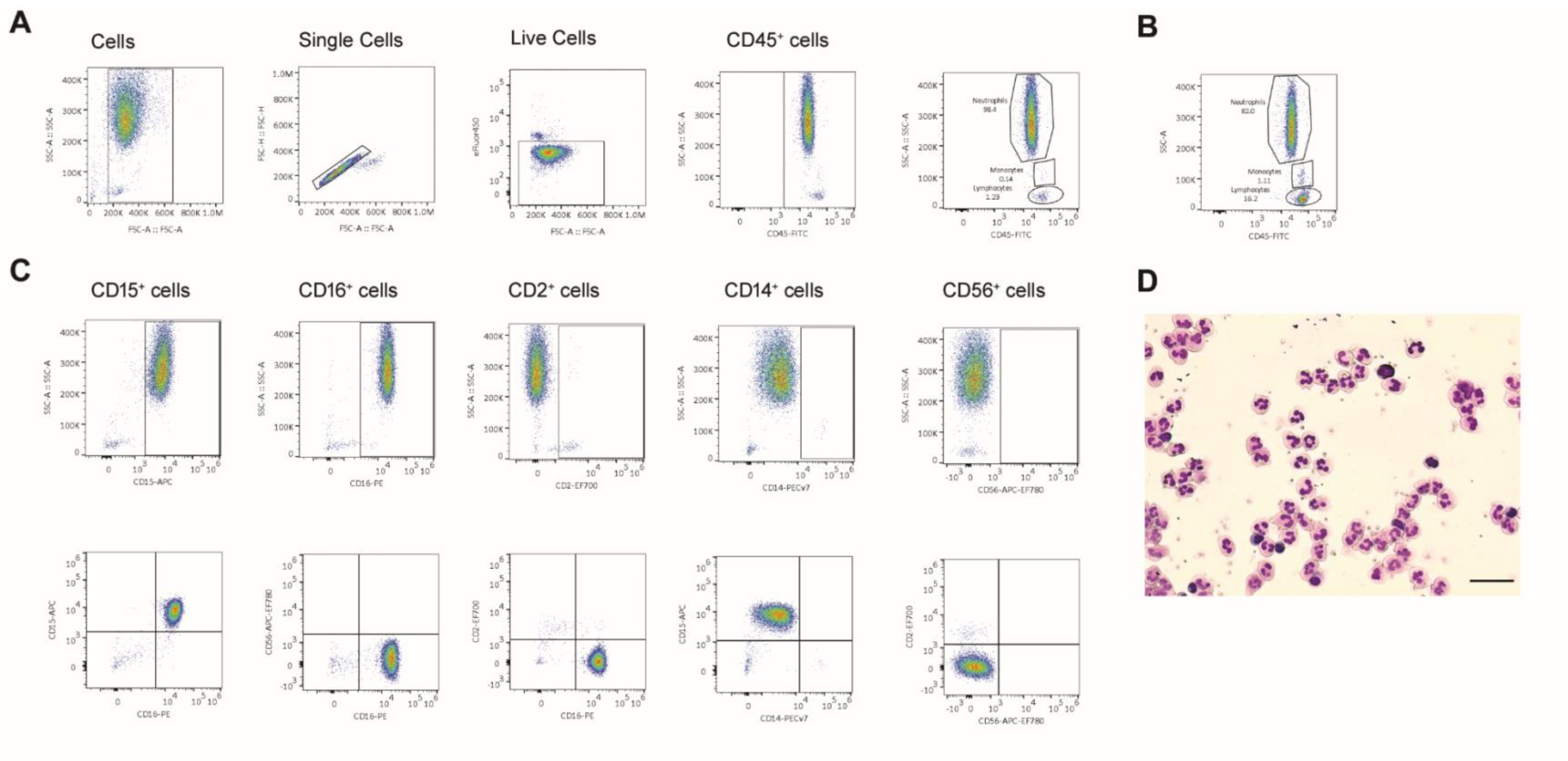

Fig. S3.

Characterization of neutrophils obtained from healthy volunteers with flow cytometry and microscopy.
The composition of the blood cell populations and the purity of the human peripheral blood neutrophils were evaluated with Flow Cytometry and microscopy before and after separation by density gradient centrifugation and lysis of erythrocytes. Gating strategy to identify and quantify lymphocytes, monocytes and neutrophils from single, live, CD45$^+$ cells was shown in a human blood sample after (**A**) and before (**B**) the neutrophils isolation. Flow cytometry analysis was also used to confirm that the purified neutrophils were CD15$^+$CD16$^+$ and CD2$^-$CD56$^-$CD14$^-$ cells (**C**). Upper row shows side scatter against the five antibodies and lower row show double staining. A representative image of the purified neutrophils is shown at 40x magnification using EVOS M5000 microscope (**D**).

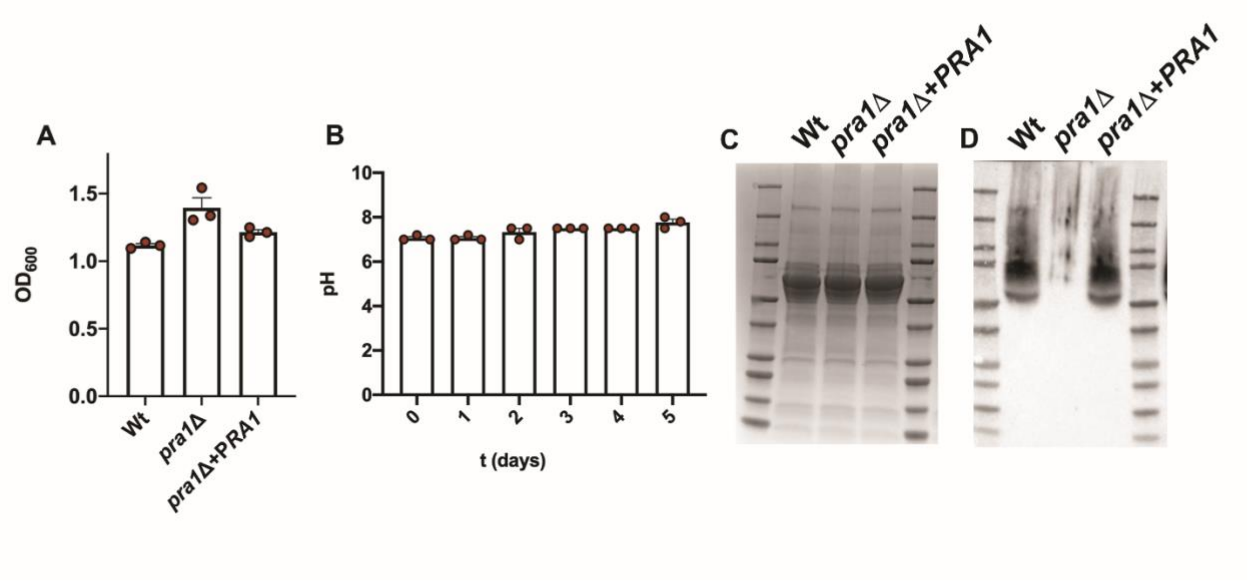

Fig. S4.

***Candida* supernatant preparation for ex-vivo neutrophils chemotaxis test**. *C. albicans* strains were cultured for five days in RPMI media during which period all strains grew to similar levels (**A**, n = 3). The pH of wild type *C. albicans* supernatant was measured daily (**B**, n = 3). Supernatant was TCA precipitated, run on a 4-20% polyacrylamide gel, and stained with Coomassie dye (**C**). Gel proteins were then transferred to a membrane and probed with polyclonal anti *C. albicans* Pra1 antibody (**D**).

A

C. glabrata BG2 + *PRA1*	
Time point	*PRA1* expression ($2^{-\Delta\Delta Ct}$)
0 h (SD)	109,396.15 ± 64,216.92
4 h	3,018.96 ± 1,220.21
18 h	3,381.95 ± 2,145
5 days	35.29 ± 5.68

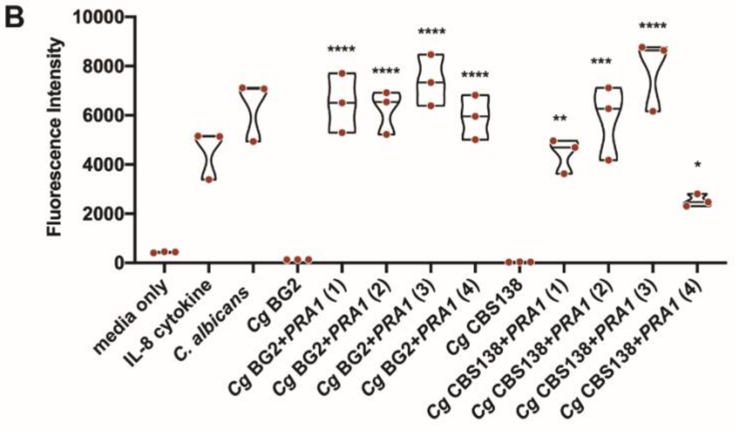

Fig. S5.

***PRA1* expression in *C. glabrata* + *PRA1* and its effect on neutrophil attraction.**

PRA1 gene expression was quantified by quantitative reverse transcription PCR at different time point in *C. glabrata* BG2 + *PRA1* strain in the SD minimal media preculture (0 h) and during incubation for 5 days in RPMI tissue culture media. The results reported in the table are the mean ± s.e.m from 3 independent experiments and the comparison to calculate $2^{-\Delta\Delta Ct}$ fold change was made using the parental *C. glabrata* BG2 strain (**A**).

After the five-day incubation period, culture filtrate of indicated strains, unconditioned media only (RPMI) or media containing the neutrophil chemotactic factor, IL-8 (100 ng/ml), were added to the lower compartment of the chemotaxis assay system. Freshly isolated human neutrophils were fluorescently labelled with Calcein and added to the upper compartment. After 2 h incubation, neutrophil chemotaxis was determined by measuring the fluorescence intensity (at 485/530 nm) in the lower compartment (n=3) (**B**).

Statistical tests in panel **B**: one way ANOVA with Dunnet post hoc test was used for comparing BG2 + *PRA1* strains vs BG2 and CBS138 + *PRA1* strains vs CBS138 (excluding positive, negative and *C. albicans* control). Error bars show the standard error of the mean. * $p < 0.05$; ** $p < 0.01$; *** $p < 0.001$; **** $p < 0.0001$.

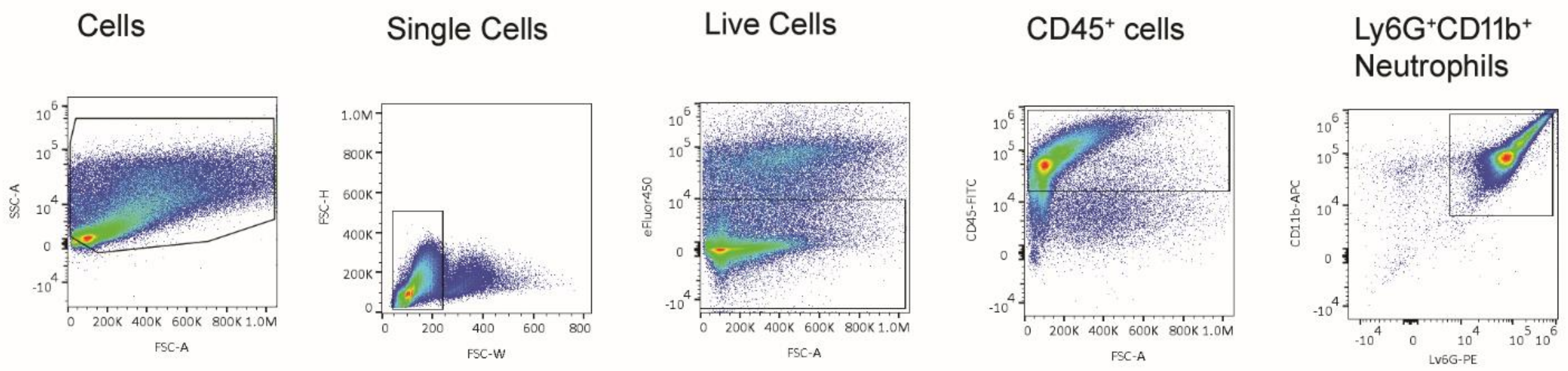

Fig. S6.

Flow cytometry gating strategy for the characterization and quantification of neutrophils in mouse vaginal lavage.
Mice were intravaginal infected with *C. albicans*. 24 h post-infection, lavage was collected and assessed for the presence of neutrophil (PMN) infiltration by flow cytometry. Gating strategy to identify and quantify Ly6G^{+}CD11b^{+} neutrophils-cells from single, live (eFluor450) CD45^{+} cells is shown from one infected mouse.

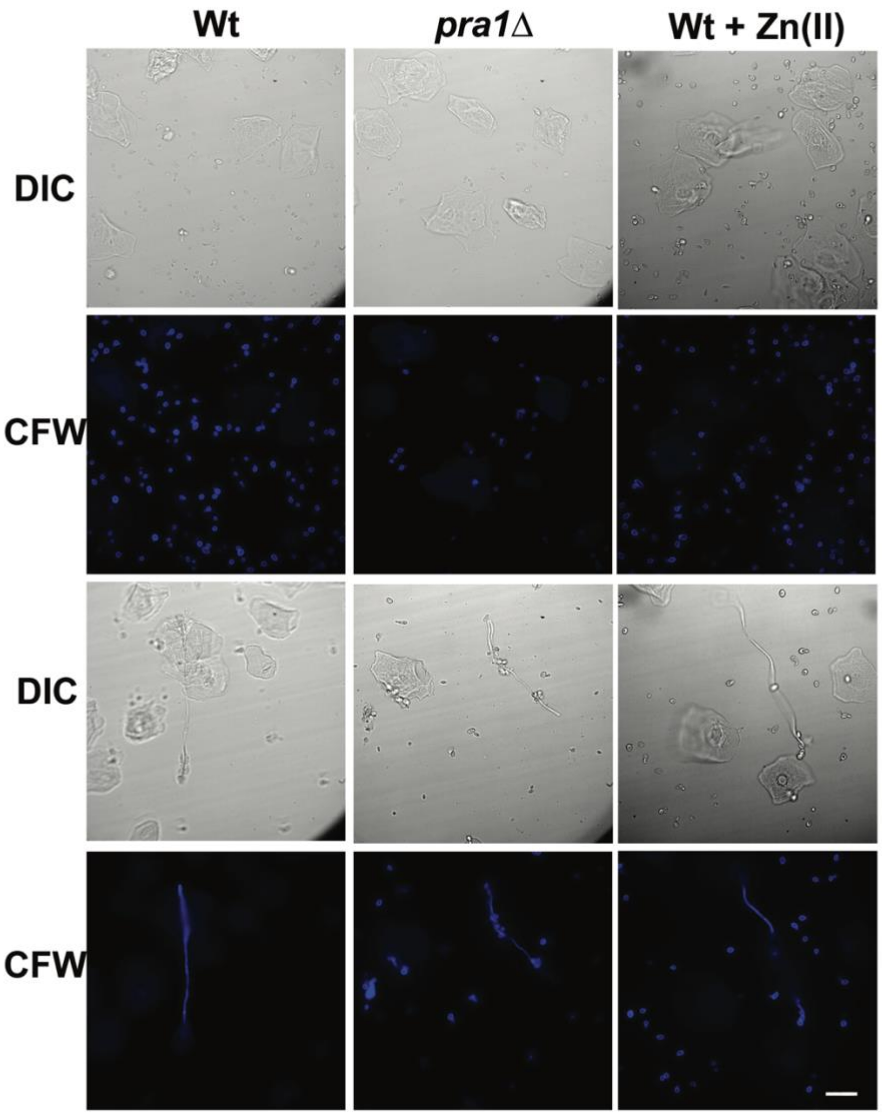

Fig. S7.
***In vivo* morphologies of *C. albicans* following vaginal infection**.
Mice were intravaginal infected with indicated *C. albicans* strains, with or without zinc (10 µl, 25 µM). 24 h post-infection, lavage was collected and stained with calcofluor white (CFW). Images show *C. albicans* cells using Differential Interference Contrast (DIC) or DAPI channel fluorescence (CFW).

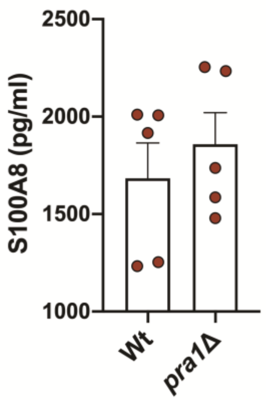

Fig. S8.
S100A8 (calprotectin) levels following infection with *C. albicans* wild type and *pra1*Δ strains.
Mice were intravaginal infected with indicated *C. albicans* strains. 24 h post-infection, lavage was collected and calprotectin assessed by measuring mouse S100A8 specific ELISA.

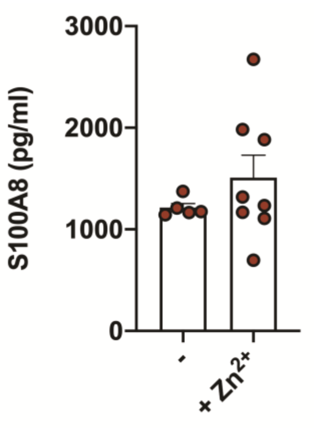

Fig. S9.
Effect of zinc on S100A8 (calprotectin) levels following *C. albicans* infection.
Mice were intravaginal infected with indicated *C. albicans* strains. 24 h post-infection, lavage was collected and calprotectin assessed by measuring mouse S100A8 specific ELISA.

ID	Diagnosis	Vaginal infection symptoms	*Candida* species	Presence of Bacterial vaginosis	Lactobacilli	PMN	Vaginal pH	Calprotectin (pg/mL)	IL-1β (pg/mL)	IL-8 (pg/mL)	*C. albicans* fungal burden (n° cells)	*PRA1* ($2^{-\Delta\Delta Ct}$)	*ECE1* ($2^{-\Delta\Delta Ct}$)	*HWP1* ($2^{-\Delta\Delta Ct}$)	*SAP6* ($2^{-\Delta\Delta Ct}$)
**V1**	VVC	asymptomatic	*C. albicans*	-	++	-	4.3	108.05	154.32	390.77	1819	42.25	0.0078	0.05	1.75
**V2**	VVC	asymptomatic	*C. albicans*	-	+	+	4.2	196.1	74.71	237.23	12882	0.0048	76.63	23.1	0.00136
**V3**	VVC	asymptomatic	*C. albicans*	-	++++	-	4.5	287.2	39.22	290	380	0.2	0.05	0.2	1.42
**V4**	VVC	asymptomatic	*C. albicans*	Aspecific altered flora	+	+	5.1	263.4	16.1	75.1	2630	0.02	0.0046	0.0045	0.2
**V5**	VVC	asymptomatic	*C. albicans*	Aspecific altered flora	++++	-	5	325.1	42.34	338.03	468	119.42	0.23	0.0036	0.076
**V6**	VVC	asymptomatic	*C. albicans*	-	+	+	4.5	92.8	222.25	435.65	16596	3.73	0.00048	5.65	4.08
**V7**	VVC	asymptomatic	*C. albicans*	-	+++	+	4.4	227	88.5	333	4168693	11.4	0.00017	1.24	2.65
**V8**	VVC	asymptomatic	*C. albicans*	-	++	-	4.4	221.3	188.3	422.77	223872	13	107	35.5	0.0021
**V9**	VVC	asymptomatic	*C. albicans*	-	+	+	4.5	277.1	38.67	292.32	114815	1.1	0.007	44.63	2.1
**RV10**	RVVC	symptomatic	*C. albicans*	-	++++	+	4.5	251.65	11.94	136.2	446683	0.23	0.1	232.32	0.3
**RV11**	RVVC	symptomatic	*C. albicans*	-	++++	+	4.8	306.4	88.1	250.6	38019	0.00034	0.0042	66.25	0.00036
**RV12**	RVVC	symptomatic	*C. albicans*	-	+++	+	4.6	194.35	171.72	431.3	41687	27.47	0.0044	32	4.2
**V13**	VVC	asymptomatic	*C. albicans*	-	+	+	4	154.15	39.94737	92.60494	11220	0.0043	0.05	240.51	0.002
**V14**	VVC	asymptomatic	*C. albicans*	-	+	+	4	388.95	193.4342	240.1852	11749	49.18	0.00075	0.02	0.1
**V15**	VVC	asymptomatic	*C. albicans*	-	+	+	4	12.75	20.15789	32.14815	20893	0.0052	214	172.44	0.0078
**V16**	VVC	symptomatic	*C. albicans*	-	+	++	5	405.9	200.9211	51.7037	10233	0.0034	3.16	14.72	0.13
**V17**	VVC	symptomatic	*C. albicans*	-	+	++	5	374.7	163.7368	391.358	8511	64	298	634.73	0.17

Table S1.

Vaginal swab samples were collected from 17 *C. albicans*-infected women from two different hospitals. Each woman was characterized for the presence of vaginal infection symptoms, presence or absence of fungi, bacteria, lactobacilli, and neutrophils and assessed for pH, calprotectin, IL-1β, IL-8, *Candida* fungal burden, and the expression of *PRA1*, *ECE1*, *SAP6,* and *HWP1* genes.

ID	Diagnosis	Vaginal infection symptoms	*Candida* species	Presence of Bacterial vaginosis	Lactobacilli	PMN	Vaginal pH	Calprotectin (pg/mL)	IL-1β (pg/mL)	IL-8 (pg/mL)
**N1**	Healthy	No symptom	-	-	++	-	*4*	264.85	3.14	53.27
**N2**	Healthy	No symptom	-	-	++++	-	*3.5*	279.4	1.07	13.28
**N3**	Healthy	No symptom	-	-	++++	-	*3.5*	196.25	1.23	14.18
**N4**	Healthy	No symptom	-	-	++	-	*3.5*	276.05	25.8	58
**N5**	Healthy	No symptom	-	-	++	-	*4*	219.25	28.42	53
**N6**	Healthy	No symptom	-	-	++	-	*4*	243.95	16.17	58
**N7**	Healthy	No symptom	-	-	++++	-	*3.5*	255.8	0	0.4
**N8**	Healthy	No symptom	-	-	+++	-	*4*	287.45	4.68	25.53
**N9**	Healthy	No symptom	-	-	+++	-	*3.5*	292.15	0	18.04
**N10**	Healthy	No symptom	-	-	++++	-	*3.5*	284	0	0
**N11**	Healthy	No symptom	-	-	+++	-	*4*	469.4	9.302632	24.03704
**N12**	Healthy	No symptom	-	-	++++	-	*4*	349.5	1.157895	2.444444

Table S2.

Vaginal swab samples were collected from 12 uncolonised women from two different hospitals. Each woman was characterized for the presence of vaginal infection symptoms, presence or absence of fungi, bacteria, lactobacilli, and neutrophils, and assessed for pH, calprotectin, IL-1β, and IL-8.

**Strain name**	**Genotype**	**Reference**	**Unique identifier (Wilson lab)**
BWP17+CIp30 isogenic "wild type"	ura3:: imm434/ura3:: imm434 his1::hisG/his1::hisG + CIp30	29	D2
*pra1*Δ	ura3:: imm434/ura3:: imm434 his1::hisG/his1::hisG *pra1*::*HIS1*/*pra1*::*ARG4* +CIp10	13	D5
*pra1*Δ+*PRA1*	ura3:: imm434/ura3:: imm434 his1::hisG/his1::hisG *pra1*::*HIS1*/*pra1*::*ARG4* +CIp10-*PRA1*	13	D6
BG2	Wild type + pAG423GPD-ccdB	30	G1
BG2+C. albicans PRA1 (1)	Wild type + pAG423GPD-ccdB +*PRA1*	This study	G2
BG2+C. albicans PRA1 (2)	Wild type + pAG423GPD-ccdB +*PRA1*	This study	G3
BG2+C. albicans PRA1 (3)	Wild type + pAG423GPD-ccdB +*PRA1*	This study	G4
BG2+C. albicans PRA1 (4)	Wild type + pAG423GPD-ccdB +*PRA1*	This study	G5
CBS138	Wild type + pAG423GPD-ccdB	31	G6
CBS138+C. albicans PRA1 (1)	Wild type + pAG423GPD-ccdB +*PRA1*	This study	G7
CBS138+C. albicans PRA1 (2)	Wild type + pAG423GPD-ccdB +*PRA1*	This study	G8
CBS138+C. albicans PRA1 (3)	Wild type + pAG423GPD-ccdB +*PRA1*	This study	G9
CBS138+C. albicans PRA1 (4)	Wild type + pAG423GPD-ccdB +*PRA1*	This study	G10

Table S3.

Strains used in this study.

Name	Sequence
ACT1 CA FOR	GCTCCAAGAGCTGTTTTCC
ACT1 CA REV	TTGGATTGGGCTTCATCAC
CEF3 CA FOR	GCTGTCAAAGCCATCTTACCAA
CEF3 CA REV	GCTCTCAAGATGGCAACTTTTTC
PRA1 CA FOR	GCCTCAAGTTCTCATCAACATAC
PRA1 CA REV	GGACTTCACCATCTGCATG
ECE1 CA FOR	GGGTATTCTTGGCAACATTCC
ECE1 CA REV	CCAGCAACAACAGAATCAATATC
ACT1 CG FOR	GTTGACCGAGGCTCCAATG
ACT1 CG REV	CACCGTCACCAGAGTCC
HWP1 CA FOR	CCGAAAGTGAAGTTACTACTGG
HWP1 CA REV	GTGTTTCAGTGGCTGGAG
SAP6 CA FOR	CGTTGGTATTGGTGGTGCTT
SAP6 CA REV	TCTGGTAGCTTCGTTGGCT

Table S4.

Candida primers used in this study.

Name	Sequence
IL1-β FOR	ACTCATTGTGGCTGTGGAGA
IL1-β REV	TTGTTCATCTCGGAGCCTGT
CxCL-2 FOR	CAAAGGCAAGGCTAACTGACC
CxCL-2 REV	CACATCAGGTACGATCCAGGC
CxCL-1 FOR	GGTGTCCCCAAGTAACGGAG
CxCL-1 REV	TTGTCAGAAGCCAGCGTTC
IL-6 FOR	CTGGGGATGTCTGTAGCTCA
IL-6 REV	CTGTGAAGTCTCCTCTCCGG
CxCL-5 FOR	CCTCCTCCGCAGAGCTAAAC
CxCL-5 REV	GAATAAGGACCTTGCCCGC
GAPDH FOR	ACGACCCCTTCATTGACCTC
GAPDH REV	CATTCTCGGCCTTGACTGTG

Table S5.

Mouse primers used in this study.
